# Thrombospondin-1 inhibits alternative complement pathway activation in antineutrophil cytoplasmic antibody-associated vasculitis

**DOI:** 10.1172/JCI180062

**Published:** 2025-05-08

**Authors:** Swagata Konwar, Sophie Schroda, Manuel Rogg, Jessika Kleindienst, Eva L. Decker, Martin Pohl, Barbara Zieger, Jens Panse, Hong Wang, Robert Grosse, Christoph Schell, Sabine Vidal, Xiaobo Liu, Christian Gorzelanny, Todor Tschongov, Karsten Häffner

**Affiliations:** 1 Department of Internal Medicine IV (Nephrology), Medical Center, Faculty of Medicine, University of Freiburg, Freiburg, Germany.; 2 Department of General Pediatrics, Adolescent Medicine and Neonatology, Faculty of Medicine, and; 3 Institute of Surgical Pathology, Faculty of Medicine, Medical Center-University of Freiburg, Freiburg, Germany.; 4 Plant Biotechnology, Faculty of Biology, University of Freiburg, Freiburg, Germany.; 5 Division of Pediatric Hematology and Oncology, Department of Pediatrics and Adolescent Medicine, Faculty of Medicine, Medical Center-University of Freiburg, Freiburg, Germany.; 6 Department of Oncology, Hematology, Hemostaseology and Stem Cell Transplantation, University Hospital RWTH Aachen, Aachen, Germany.; 7 Center for Integrated Oncology (CIO), Aachen, Bonn, Cologne, Düsseldorf (ABCD) Germany, Aachen, Germany.; 8 Institute of Experimental and Clinical Pharmacology and Toxicology, Medical Faculty,; 9 Centre for Integrative Biological Signalling Studies-CIBSS, and; 10 Freiburg Institute for Advanced Studies (FRIAS), University of Freiburg, Freiburg, Germany.; 11 Department of Dermatology and Venereology, University Medical Center Hamburg-Eppendorf, Hamburg, Germany.

**Keywords:** Immunology, Inflammation, Vascular biology, Complement, Endothelial cells, Vasculitis

## Abstract

Complement activation is a relevant driver in the pathomechanisms of vasculitis. The involved proteins in the interaction between endothelia, complement, and platelets in these conditions are only partially understood. Thrombospondin-1 (TSP-1), found in platelet α-granules and released from activated endothelial cells, interacts with factor H (FH) and vWF. However, to our knowledge, direct regulatory interaction with the complement cascade has not yet been described. Our study shows that TSP-1 is a potent, FH-independent inhibitor of the alternative complement pathway. TSP-1 binds to complement proteins and inhibits cleavage of C3 and C5 and the formation of the membrane attack complex. We validated complement-regulatory function in blood samples from patients with primary complement defects. The physiological relevance of TSP-1 was demonstrated in patients with antineutrophil cytoplasmic antibody-associated vasculitis (AAV) by significantly enhanced TSP-1 staining in glomerular lesions and increased complement activity and NETosis after TSP-1 deficiency in an in vitro and in vivo model of AAV. The complement-inhibiting function of TSP-1 represents an important mechanism in the interaction of endothelia and complement. In particular, the interplay between released TSP-1 and the complement system locally, especially on surfaces, influences the balance between complement activation and inhibition and may be relevant in various vascular diseases.

## Abstract 2

Thrombospondin-1 inhibits the complement cascade independently of factor H, revealing a significant role in vasculitis pathomechanisms and potential therapeutic implications.

## Introduction

Thrombospondins (TSPs) are multifunctional, matricellular glycoproteins consisting of 5 members (TSP-1, TSP-2, TSP-3, TSP-4, and TSP-5) (1). TSP-1 is the first and most prominent member of this protein family. It is the major component of the α-granules in platelets (up to 25%) and can additionally be released from the Weibel-Palade bodies of endothelial cells (2, 3). TSP-1 is also produced and secreted after tissue injury by a variety of cells, including endothelial cells, fibroblasts, and macrophages (4). TSP-1 is involved in the regulation of clot formation and platelet aggregation (5). Furthermore, TSP-1 stabilizes platelet aggregates by inhibiting vWF-mediated proteolysis by ADAMTS13 (6, 7). TSP-1 supports wound healing through the activation of latent TGF-β and is involved in fibrotic diseases like diabetic nephropathy, liver fibrosis, and multiple myeloma (8). It also has been shown to be a potent inhibitor of the NO signaling cascade through its interaction with the cell surface receptor CD47 (9).

Until now, complement regulatory functions have only been shown for TSP-5 in rheumatoid arthritis, inhibiting the classical pathway (10). Considering the similar structure of all thrombospondins, other members of this family could also possess complement regulatory functions. In particular, TSP-1 is likely to interact with the complement system because of its presence in platelets and endothelial cells. In addition, TSP-1 has been shown to co-purify with complement factor H (FH), the most important regulator of the alternative complement system, isolated from platelets (11). Surface plasmon resonance (SPR) studies have shown that TSP-1 can bind to FH with high affinity, thereby increasing the binding of FH to platelets (12).

Defects in alternative complement pathway regulation can predispose to the onset of several severe diseases (13). In atypical hemolytic uremic syndrome (aHUS), complement overactivation on endothelial surfaces, oftentimes caused by FH mutations, promotes thrombotic microangiopathy and consequently hemolytic anemia, thrombocytopenia, and acute and chronic renal failure (14). In paroxysmal nocturnal hemoglobinuria (PNH), acquired mutations in the phosphatidylinositol glycan class A gene on hematopoietic stem cells lead to a loss of CD55 and CD59, important negative complement regulators on red cell membranes (15). Consequently, these erythrocytes are not sufficiently protected from complement-mediated lysis. Although some patients are only affected by mild anemia, a substantial number of untreated patients develop blood clots that can result in life-threatening thromboembolic events and represent the leading cause of death from this condition (16).

As these direct complement-driven diseases demonstrate, complement activation on surfaces leads to an interplay between the endothelium, complement, platelets, and the coagulation system (17–20). In addition, emerging studies from basic and clinical research show an interaction between these systems even in nonprimary complement diseases, for example, antineutrophil cytoplasmatic antibody-associated vasculitis (AAV) (21).

In AAV, patients develop autoantibodies against neutrophil antigens that cause inflammation, infiltration of immune cells, and necrosis of small blood vessels (22). Animal models (23) have demonstrated a clear link between vascular inflammation and alternative pathway complement activation, which led to the development and clinical implementation of the C5a receptor inhibitor avacopan (24).

A deeper understanding of the proteins involved in this interplay between endothelium, complement, and platelets could lead to the identification of new pathomechanisms and generate new therapeutic strategies to improve patient health and survival. Considering the relationship of TSP-1 to FH and its involvement in inflammation, wound healing, and thrombosis, we investigated the ability of TSP-1 to directly interact with the complement system. We examined binding of TSP-1 to alternative pathway complement proteins besides FH and deciphered its complement regulatory functions in vitro. The complement inhibitory functions of TSP-1 were further verified in ex vivo models using blood samples from healthy individuals and patients with AAV and in an in vivo mouse model with complement overactivation.

The interaction of TSP-1 and the complement system was investigated in stimulated endothelial cells, where the lack of TSP-1 increased complement C3 deposition and endothelial activation. In patients with AAV, the relevance of TSP-1 regarding disease activity was examined and confirmed in TSP-1–deficient (TSP-1-KO) mice. These investigations provide evidence for an additional local complement regulatory function of TSP-1, especially in vasculitis, representing one important player in these conditions, which opens up potentially new aspects for therapeutic interventions.

## Results

### TSP-1 has an FH-independent complement-regulatory function on the alternative complement pathway.

**Figure 1:**
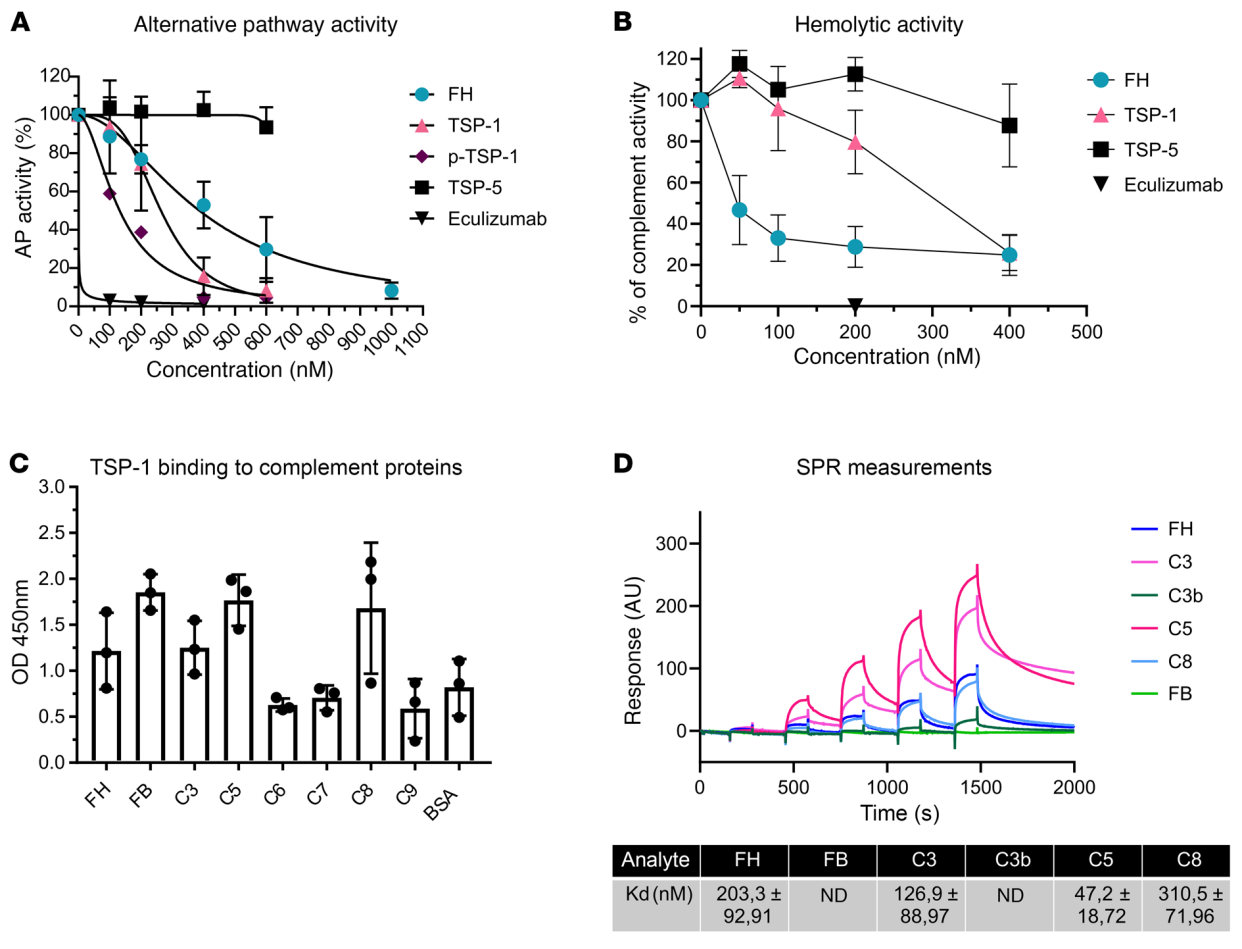
TSP-1 has distinct complement regulatory roles compared with TSP-5, protects sheep erythrocytes from complement-mediated hemolysis independent of FH, and binds to central complement proteins of the alternative pathway. (**A**) TSP-1 inhibits alternative complement pathway activation in contrast to TSP-5. The alternative complement pathway in NHS was activated on LPS-coated wells and with increasing concentrations of FH, eculizumab, TSP-1, or TSP-5. Platelet-derived TSP-1 (p-TSP-1) was used as an additional control to exclude artifacts caused by the histidine tag used for purification. (**B**) TSP-1 protects sheep erythrocytes from alternative complement pathway–mediated lysis in the absence of FH. Sheep erythrocytes were incubated with FH-depleted serum and increasing concentrations of FH, TSP-1, or TSP-5. Data are shown as mean ± SD. The alternative complement pathway and hemolytic activity were normalized against untreated control samples. Data were fitted using nonlinear regression. (**C**) TSP-1 binds to central proteins of the alternative pathway. Complement proteins FH, FB, C3, C5, C6, C7, C8, C9, or BSA were coated on microtiter plates and incubated with recombinant TSP-1. Bound TSP-1 was determined using specific antibodies. (**D**) Surface plasmon resonance (SPR) Biacore measurements demonstrating the binding of TSP-1 to key proteins of the alternative complement pathway. TSP-1 was immobilized on CM5 chips at a concentration of 0.1 μM. Binding interactions with complement proteins FH, FB, C3, C3b, C5, and C8 were assessed at various concentrations (12.3, 37.03, 111.1, 333.3, 1,000 nM). The binding data were fitted using a 1:1 Langmuir binding model to determine on and off rates, which were then used to calculate affinity constants (Kd). The graph depicts a summary of the binding of complement proteins to TSP-1 at increasing concentrations. Average Kd values, calculated from 3 repeated measurements, are presented in the accompanying table. Data are shown as mean ± SD of 3 independent experiments. ND, not detected.

We used recombinant full-length TSP-1 and elucidated its ability to inhibit the alternative complement pathway by ELISA in normal human serum. Complement activation was assessed using microtiter plates coated with specific activators of the alternative complement pathway and detecting the amount of membrane attack complexes (MACs) formed in the wells. TSP-1, but not TSP-5, showed pronounced inhibition of the alternative complement pathway at concentrations above 400 nM (Figure 1A). To ensure that the observed experimental effect was not influenced by the histidine tag of recombinant TSP-1, platelet-isolated TSP-1 (p-TSP-1) was also employed in this experiment, yielding comparable results (Figure 1A).

The significance of the interaction between TSP-1 and FH was assessed through a hemolytic assay involving sheep erythrocytes and FH-depleted human serum, which leads to hemolysis of the erythrocytes due to FH deficiency. TSP-1, in contrast to TSP-5, was able to reduce hemolysis (Figure 1B). The inhibition of hemolysis reached approximately 70% at concentrations of 400 nM. The fact that TSP-1 could suppress hemolysis in FH-depleted serum implies that TSP-1’s inhibition of the alternative complement pathway is, to some extent, independent of FH.

### TSP-1 binds to central complement proteins of the alternative pathway.

In order to explore potential interactions between TSP-1 and the complement system, we investigated the binding of TSP-1 to alternative complement pathway proteins using ELISA. Our findings revealed that TSP-1 bound to FH, factor B (FB), C3, C5, and C8 (Figure 1C). To validate these interactions, binding ELISAs were conducted with platelet-isolated TSP-1, eliminating potential binding artifacts introduced by the histidine tag of the protein (Supplemental Figure 1; supplemental material available online with this article; https://doi.org/10.1172/JCI180062DS1). For a comprehensive understanding of binding affinities, we performed SPR Biacore measurements for TSP-1 with FH, FB, C3, C3b, C5, and C8. The strongest binding was observed with C5 (47 nM), followed by C3 (127 nM), FH (203 nM), and C8 (311 nM) (Figure 1D). Although binding to FB and C3b was noted, the affinity was insufficient to determine Kd values within our tested concentration ranges. Since TSP-1 interacts with several alternative pathway proteins, we aimed to gain a deeper understanding of the feasibility and mechanism of these interactions. To achieve this, we utilized AlphaFold 3 predictions. For both FH and C3, the analysis predicted that type 1 and type 2 repeats play a role in protein-protein interactions (Supplemental Figure 3, A–C). Additionally, for C3, the N-terminal domain, procollagen domain, and type 1 repeats were also implicated in binding. Notably, these domains are exclusive to TSP-1 and TSP-2 and are absent in all other thrombospondins, suggesting that interactions with the alternative pathway are unique to these 2 proteins. Given these interactions with several alternative complement pathway proteins, we subsequently employed several in vitro assays to specifically assess TSP-1–dependent complement inhibition.

### TSP-1 inhibits factor D–mediated cleavage of FB.

**Figure 2:**
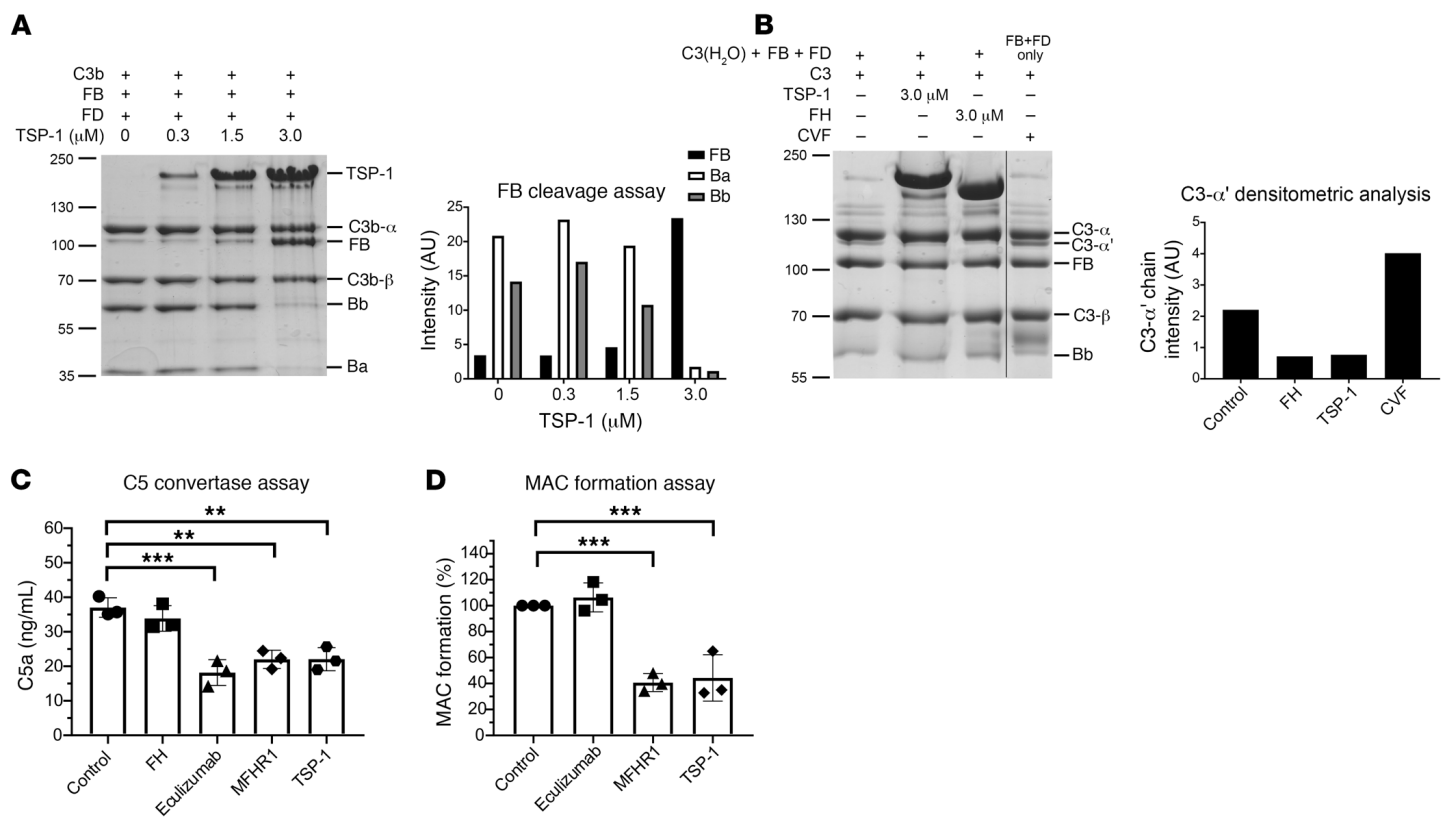
TSP-1 modulates complement at the C3 and C5 level of the complement cascade. (**A**) TSP-1 protects FB from cleavage by FD. FB was incubated with FD, C3b, and varying concentrations of TSP-1. Subsequently, FB and its cleavage products were visualized by Coomassie blue staining. Graph on the right illustrates band intensity of FB and its cleavage products. (**B**) TSP-1 inhibits cleavage of C3 by the alternative complement pathway C3 convertase. C3 convertase was generated by incubating C3(H2O) with FB and FD, followed by the addition of C3 in the presence or absence of TSP-1 or FH. As a positive control, CVF C3 convertase (CVF + FB + FD) was utilized. The graph on the right depicts the band intensity of C3-α′ chain. Lanes were run on the same gel but were noncontiguous. Data are shown as mean ± SD. (**C**) TSP-1 inhibits cleavage of C5. Cobra venom factor (CVF) convertase (CVFBb) was generated and added to C5 preincubated with FH, eculizumab, MFHR1, or TSP-1. The amount of released C5a was quantified by ELISA. Data are shown as mean ± SD. ANOVA was used with Dunnett’s multiple-comparison test. (**D**) TSP-1 inhibits the formation of MAC. Sheep erythrocytes were premixed with C7 (9 nM), C8 (7 nM), and C9 (15 nM). BSA, eculizumab, MFHR1, or TSP-1 (1.3 μM each) were preincubated with C5b-6 (0.7 nM) and then added to the erythrocytes. Data are shown as mean ± SD of 3 independent experiments; **P ≤ 0.01, ***P ≤ 0.01; 1-way ANOVA was used with Tukey’s post hoc test for comparison against control.

Complement activation involves the cleavage of FB by factor D (FD) in the presence of C3b to build up C3 convertase. Therefore, we investigated whether TSP-1 could inhibit the cleavage of FB. C3b was incubated with FB, FD, and increasing amounts of TSP-1. Without TSP-1, almost all FB (93 kDa) underwent cleavage by FD into Ba (33 kDa) and Bb (60 kDa). However, as the concentration of TSP-1 increased, a pronounced decrease in the cleavage products of FB was observed (Figure 2A). This finding indicates that TSP-1 interacts with and protects FB from FD-mediated cleavage.

### TSP-1 prevents the cleavage of C3 by C3 convertase.

We examined whether TSP-1 influences the complement system by acting on FH, the primary regulator of the alternative pathway. Our findings revealed that TSP-1 does not affect the decay-acceleration or cofactor functions of FH (Supplemental Figure 2, A–D). Furthermore, TSP-1 does not possess intrinsic decay-acceleration or cofactor activity. To determine the ability of TSP-1 to influence C3 cleavage, C3 convertase was generated by incubating C3(H_2_O) with FB and FD. C3 was then added to the reaction in the presence or absence of TSP-1 or FH, and the amount of cleaved C3 products was visualized. Cobra venom factor (CVF) was used as a positive control. C3 is a 185 kDa protein consisting of a C3-α chain (110 kDa) and a C3-β chain (75 kDa) linked by 2 disulfide bonds. The C3 convertase cleaves the C3-α chain into C3a (9 kDa) and the C3-α′ chain (101 kDa). A pronounced C3-α′ chain was observed in the presence of CVF and in the control sample without any inhibitor (Figure 2B). TSP-1 or FH reduced the amount of generated C3b-α′ chain compared with the control sample, indicating that both proteins inhibited the cleavage of C3.

### TSP-1 prevents the cleavage of C5 and the formation of the MAC.

To assess the functional relevance of the interaction between TSP-1 and C5, we employed a CVF convertase assay. It involved the incubation of C5 in the presence or absence of FH, eculizumab, TSP-1, or MFHR1 (a recombinant FH-FHR1 fusion protein known for inhibiting C5 cleavage and MAC formation) (25). The quantification of produced C5a was carried out using ELISA. Unlike FH, TSP-1 was able to reduce C5a generation, similarly to eculizumab and MFHR1. This suggests that TSP-1 is able to protect C5 from cleavage into C5a and C5b (Figure 2C). MAC formation marks the endpoint of the complement cascade. Given the interaction of TSP-1 with C8, we investigated whether TSP-1 could directly influence MAC formation beyond inhibiting C5 cleavage. MAC forms when C5b is present on surfaces along with C6–C9. To explore TSP-1’s potential inhibition of direct MAC formation, TSP-1 was mixed with C7, C8, and C9 and then added to sheep erythrocytes preincubated with C5b. In contrast to eculizumab, TSP-1 demonstrated a robust inhibition of MAC formation and lysis of sheep erythrocytes, comparable to MFHR1 as a positive control (Figure 2D) (25).

### TSP-1 inhibits hemolytic activity and C3 deposition on endothelial cells in aHUS sera.

**Figure 3:**
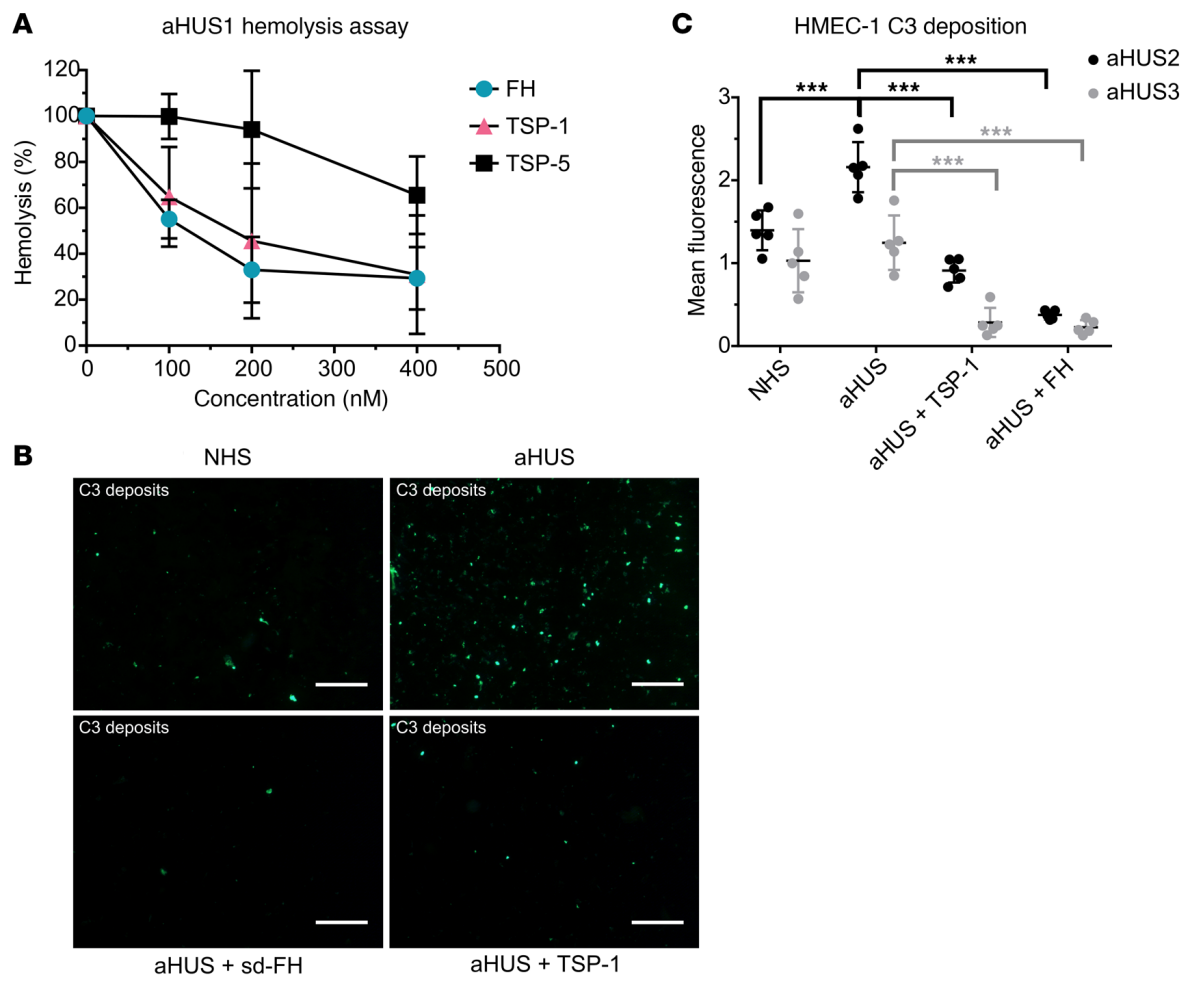
TSP-1 inhibits hemolytic activity and pathogenic C3 deposition on endothelial cells when added to the serum of patients with aHUS. (**A**) TSP-1 protects sheep erythrocytes from complement-induced lysis in aHUS1 serum. Sheep erythrocytes were incubated with aHUS1 serum and increasing concentrations of FH, TSP-1, or TSP-5. Data were normalized against erythrocytes treated with aHUS1 serum without inhibitors. Data are shown as mean ± SD of 3 independent experiments. (**B**) Representative fluorescence images of C3 deposits on HMEC-1 cells treated with aHUS sera. HMEC-1 cells were activated with ADP and incubated with 50% normal human serum or aHUS serum with or without 1 μM TSP-1 or FH and stained for C3 deposits. (**C**) TSP-1 prevents C3 deposition on endothelial cells treated with aHUS sera. Mean fluorescence analysis showed that aHUS2 and aHUS 3 serum caused strong deposition of C3 molecules on HMEC-1, which could be prevented by addition of either FH or TSP-1 into the serum. C3 fluorescence intensity was measured in at least 5 randomly chosen high power fields. Data are shown as mean ± SD of 5 independent experiments. ***P ≤ 0.01; 1-way ANOVA was used with Tukey’s multiple-comparison test. Scale bar: 100 μm.

Serum from a healthy sibling (aHUS1) belonging to a family with aHUS and carrying a known FH mutation for aHUS (R1215Q, SCR20) (patients’ details in Supplemental Table 1) was used in a hemolytic assay. The FH mutation causes strong hemolytic activity when incubated with sheep erythrocytes. TSP-1, in contrast to TSP-5, was able to reduce hemolysis when added to the aHUS1 serum, as was FH. More than 70% of hemolysis could be inhibited at concentrations of around 200 nM TSP-1 (Figure 3A). We further confirmed the ability of TSP-1 to protect erythrocytes from complement C3 deposits by using human PNH erythrocytes, which are susceptible to complement-mediated lysis due to their lack of cell surface complement receptors (Supplemental Figure 4).

Endothelial injury and complement deposition on endothelial cells are a hallmark of the pathology of complement-mediated diseases like aHUS. To investigate whether TSP-1 could influence complement deposition on endothelial cells, a cell-based assay involving human microvascular endothelial cells (HMEC-1) was performed (26). In this assay, sera from patients with aHUS were incubated on ADP-stimulated HMECs, and the amount of C3 deposition was analyzed by immunofluorescence staining. The use of sera from 2 patients with known aHUS characteristics (aHUS2 and aHUS3, Supplemental Table 1) resulted in increased C3 deposition on HMECs compared with normal human serum. In contrast, supplementation of the sera with TSP-1 or FH led to a significant reduction in C3 deposition (Figure 3, B and C). Using a similar experimental approach, we investigated whether TSP-1 binding to the cell surface is sufficient to inhibit C3 deposition on endothelial cells. The cells were first incubated with TSP-1, followed by 3 wash steps before exposure to aHUS serum. Preincubation with TSP-1 effectively prevented pathological C3 deposition on endothelial cells, comparable to its direct addition into the supernatant (Supplemental Figure 5). These findings suggest that TSP-1 can regulate complement activity at surfaces where its local concentration is elevated.

### Knockdown of TSP-1 significantly increases deposition of C3 on activated endothelial cells.

**Figure 4:**
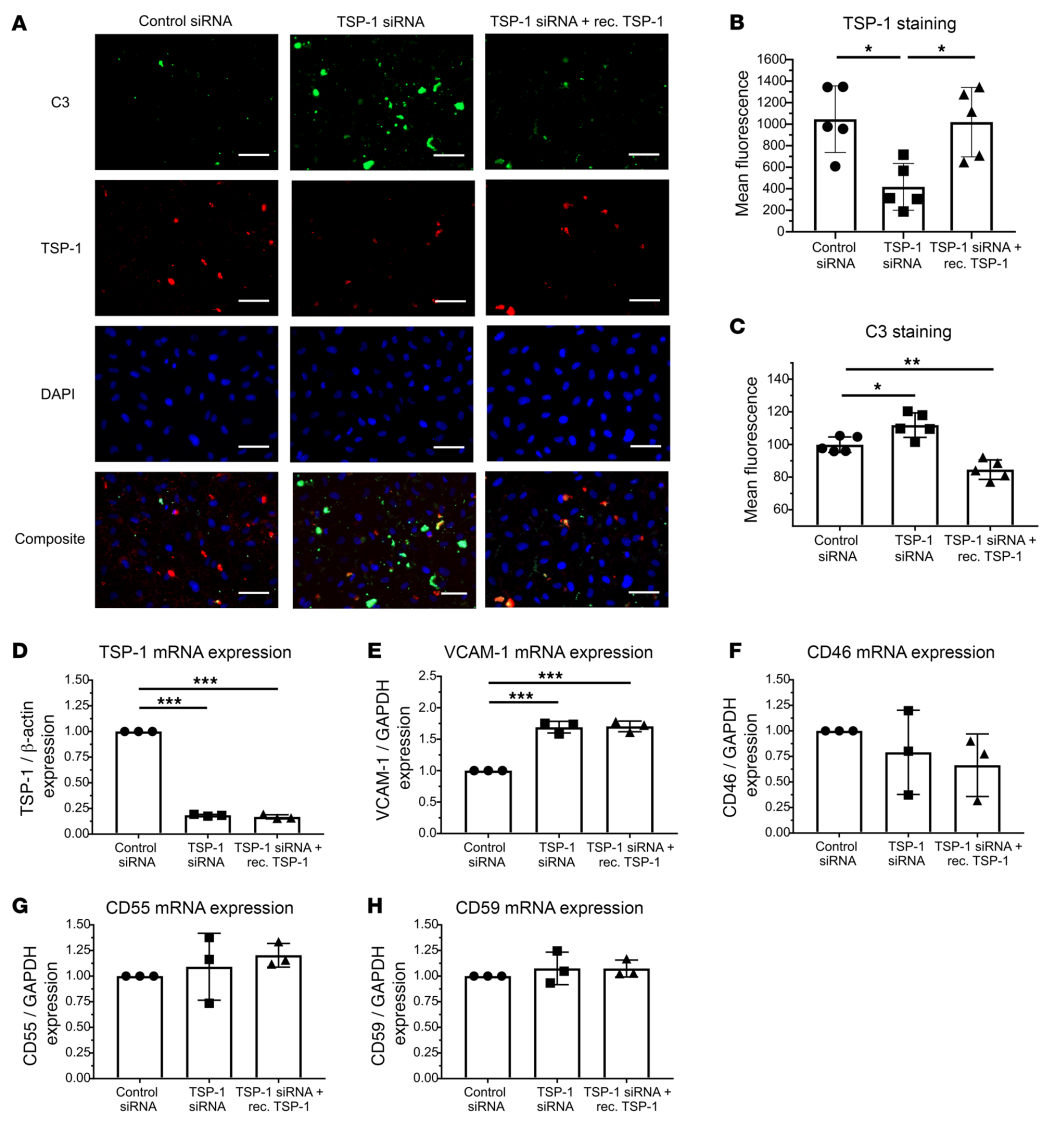
Knockdown of TSP-1 significantly increases deposition of C3 on activated endothelial cells. (**A**) Representative images showing reduced TSP-1 levels and increased C3 deposition in HUVECs after TSP-1 siRNA treatment. Addition of recombinant TSP-1 led to a significant reduction in C3 deposition. (**B** and **C**) Analysis of mean fluorescence of TSP-1 and C3 staining intensity on siRNA-treated HUVECs. Data are presented as mean ± SD of 5 independent experiments. (**D**) qPCR analysis confirming greater than 80% efficiency of TSP-1 knockdown in HUVECs. Data are presented as mean ± SD from 3 independent experiments. (**E**) VCAM-1 mRNA expression was significantly elevated in TSP-1–deficient cells compared with controls. Data are shown as mean ± SD from 3 independent experiments. (**F**–**H**) Expression of complement regulatory proteins CD55, CD59, and CD46 was assessed, with no significant changes observed for CD55 and CD59, while CD46 expression showed a nonsignificant trend toward reduction. Data are shown as mean ± SD of 3 independent experiments. *P ≤ 0.05, **P ≤ 0.01, ***P ≤ 0.001; 1-way ANOVA was used with Tukey’s multiple-comparison test. Scale bar: 100 μm.

Given that TSP-1 is stored in Weibel-Palade bodies of endothelial cells, we investigated its role in complement regulation. HUVECs were chosen for this study because of their higher TSP-1 production and secretion compared with HMEC-1 cells (Supplemental Figure 6). To suppress TSP-1 expression, HUVECs were treated with TSP-1–specific siRNA and stimulated with histamine to activate endothelial cells. Knockdown efficiency was confirmed by qPCR, which showed over 80% reduction in TSP-1 mRNA (Figure 4D), and fluorescence analysis indicated a 40%–60% decrease in surface TSP-1 protein levels (Figure 4B). Supplementing with exogenous TSP-1 reduced C3 deposits, confirming its protective function (Figure 4, A and C). We further examined whether TSP-1 depletion altered the expression of markers related to endothelial activation and complement regulation. VCAM-1, a marker of endothelial activation, was markedly upregulated in TSP-1–deficient cells, and this increase could not be attenuated by recombinant TSP-1 (Figure 4E). However, it is important to note that changes in VCAM-1 expression typically require several hours to develop (27, 28). Therefore, a reduction in VCAM-1 expression was not expected within the experimental time frame of 1 hour. In contrast, the expression levels of the complement regulatory proteins CD55 and CD59 were unaffected, whereas CD46 showed a minor but nonsignificant reduction (Figure 4, F–H).

These findings demonstrated that TSP-1 plays a key role in protecting endothelial cells from complement-mediated damage by limiting C3 deposition. However, its deficiency did not significantly alter the expression of major complement regulatory proteins, suggesting a direct mechanism of complement control.

### TSP-1 modulates complement activity in an in vivo mouse model of primary complement overactivation.

**Figure 5:**
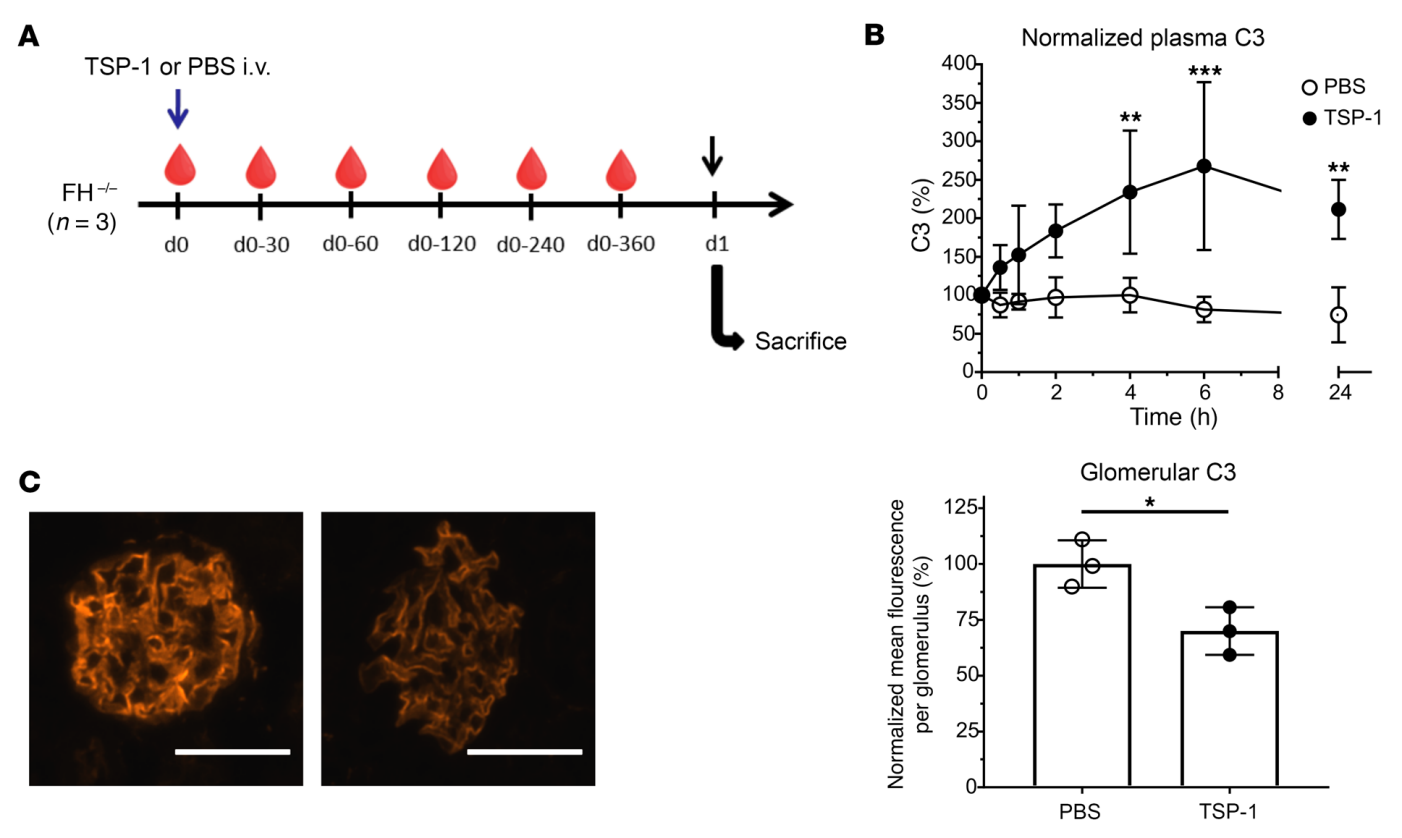
TSP-1 regulates complement activation in FH-deficient mice. (**A**) FH-deficient mice were injected with TSP-1 or PBS (*n* = 3 each), followed by serial blood sampling and kidney collection for histological analysis. (**B**) Serum C3 levels increased within 30 minutes after injection, peaked at 6 hours, and remained elevated until 24 hours after injection compared with PBS-treated mice. (**C**) Representative images of glomerular C3 and analysis of glomerular C3 deposits of FH-deficient mice 24 hours after injection of TSP-1. TSP-1 injection led to a significant reduction in glomerular C3 deposits. Data are shown as mean ± SD; statistical analysis was performed using 2-way ANOVA for plasma C3 concentrations and Student’s *t* test for analysis of glomerular C3 deposits. *P ≤ 0.05, **P ≤ 0.01, ***P ≤ 0.001. Scale bar: 40 μm.

To assess the in vivo role of TSP-1 in the absence of FH, we used FH-deficient mice, an established model for complement overactivation, characterized by low serum C3 levels and extensive C3 deposition within the kidneys, resembling C3 glomerulopathy (29). Mice were injected with 400 μg of TSP-1, and blood samples were collected at multiple time points over 24 hours. Kidneys were harvested for histological examination (Figure 5A).

TSP-1 administration led to an increase in serum C3 levels, detectable within 30 minutes after injection and peaking around 6 hours (Figure 5B). Serum C3 levels were significantly increased compared with FH-deficient mice 6 hours after injection, and increased levels lasted up to 24 hours. Correspondingly, a significant reduction in glomerular C3 deposits was observed 24 hours after TSP-1 injection (Figure 5C). These findings demonstrated that TSP-1 can modulate complement activity at the C3 level independently of FH in vivo.

### Substantial TSP-1 staining in AAV renal biopsies.

**Figure 6:**
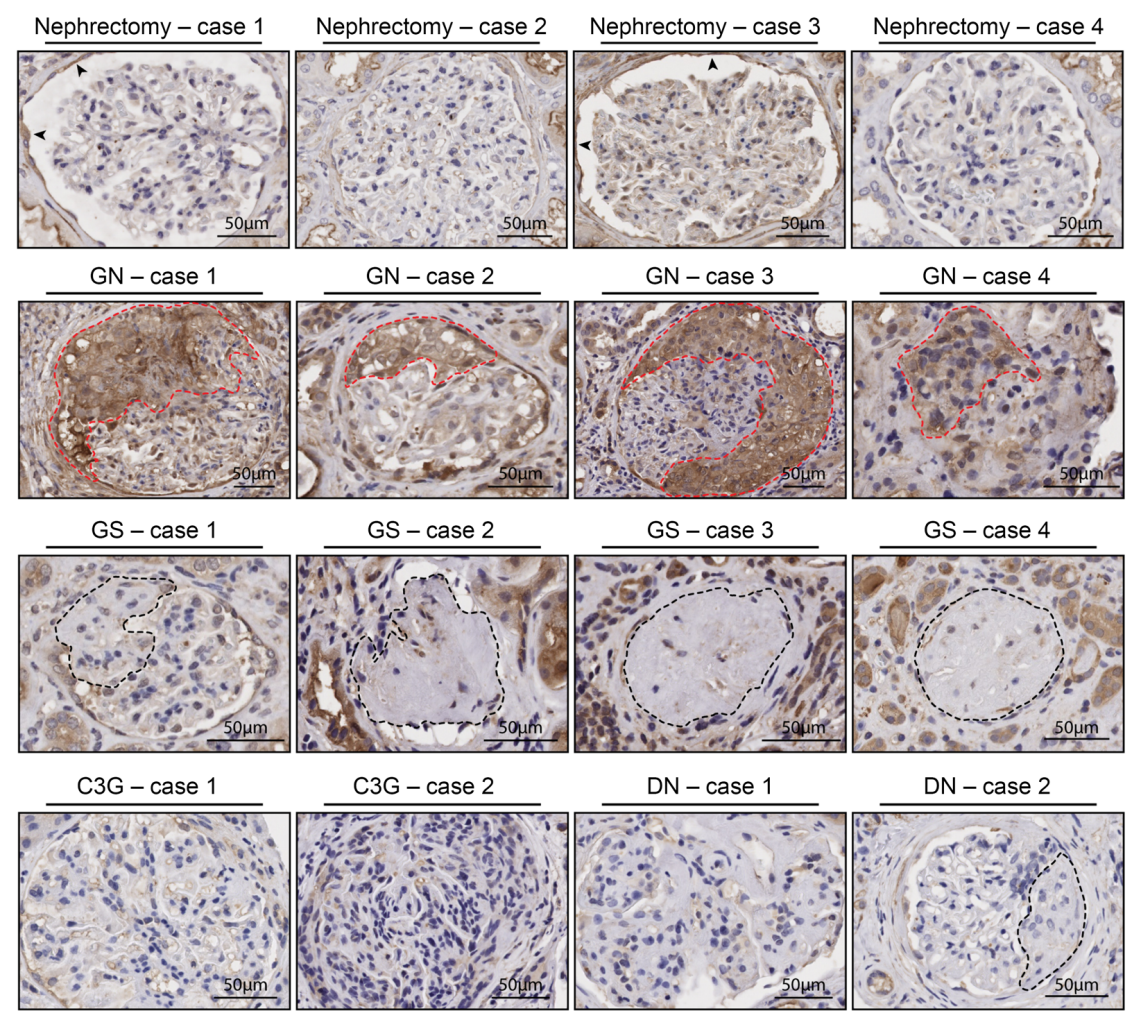
TSP-1 markedly expressed in glomerular crescents of patients with AAV. Representative images of TSP-1 IHC staining of kidney biopsies and cancer nephrectomies. Pronounced TSP-1 staining can be seen in glomerular crescents (red dashed lines) of 4 patients with AAV (GN), whereas TSP-1 is absent in unaffected samples of cancer nephrectomies, patients with focal segmental glomerulosclerosis (GS; black dashed lines indicate sclerosis), C3 glomerulopathy (C3G), or diabetic nephropathy (DN). Scale bar: 50 μm.

Recent publications have reported significantly increased TSP-1 plasma levels in patients with AAV (30, 31). Considering that complement activation is a driving force of neutrophil extracellular trap (NET) release and that TSP-1 was able to protect endothelial cells from complement mediated injury in our experiments, we elucidated whether the complement-regulating function of TSP-1 could potentially influence this process. To evaluate a (patho-)physiological involvement of TSP-1 in AAV, we stained kidney biopsies from 4 patients with AAV (patient details in Supplemental Table 3). TSP-1 showed strong positivity in all glomerular crescent lesions observed in AAV biopsies (Figure 6). This was in contrast to unaffected tumor-nephrectomy control samples and biopsies from patients with focal segmental sclerosis, who also had renal impairment, hematuria, and proteinuria. We also investigated TSP-1 staining in C3 glomerulopathy, a primary complement-mediated disease, and in diabetic nephropathy. No enhanced staining could be detected, particularly in disease-specific lesions in these pathologies.

### In vitro vasculitis model to investigate the role of TSP-1 in AAV.

Building upon previous findings, we hypothesized that TSP-1 regulates NET formation by modulating complement activity. To test whether TSP-1 can regulate NET formation, we employed a microfluidic perfusion model using HUVECs. To preserve complement activity, hirudin-treated blood from healthy volunteers was collected and stained with DAPI to visualize NET release. As controls, blood samples were either left untreated or stimulated with PMA, a potent inducer of NETosis. To mimic the conditions of AAV, proteinase 3 (PR3) antibodies isolated from a patient (250 U/mL, equivalent to the patient’s blood concentration) were added. TNF-α is used as a stimulus to induce neutrophil priming, a key mechanism in AAV-mediated NETosis. Since PR3 antibodies alone are insufficient to trigger visible NETosis, TNF-α was used as a secondary stimulus (32, 33).

To investigate TSP-1’s role in modulating complement-mediated NETosis, recombinant TSP-1 was added to samples treated with PR3 and TNF-α. Conversely, to simulate TSP-1 deficiency, a blocking antibody against TSP-1 (A6.1) was used in blood treated with PR3 only. Our hypothesis was that TSP-1 deficiency might act as a secondary trigger, promoting complement activation and NET release. To ensure that the observed effects were specifically due to TSP-1 blockade and not nonspecific effects from the antibody itself, separate experiments were performed using an isotype control antibody (Supplemental Figure 7). Live-cell imaging was used to monitor NET formation over 4 hours, and supernatants were collected to measure TSP-1, complement C5a, and DNA-histone complexes. Endothelial cells were fixed and stained to assess complement C3 deposition and VCAM-1 expression as a marker of endothelial activation.

**Figure 7:**
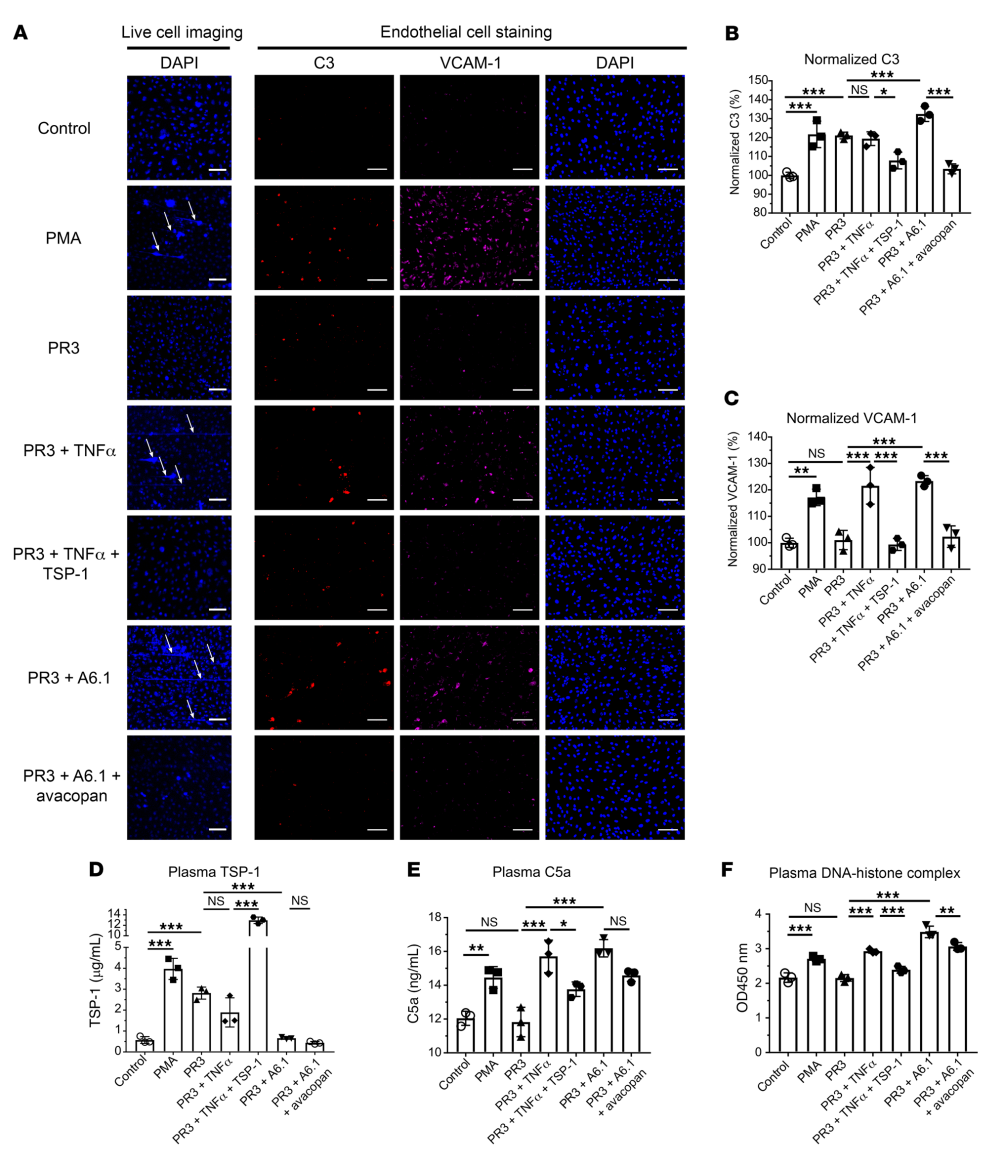
TSP-1 regulates complement activation and NET formation in an in vitro model of AAV. (**A**) Representative live-cell imaging showing NET formation in microfluidic perfusion experiments with HUVECs cultured on μ-slides. Untreated control samples exhibited no NET formation, minimal complement C3 deposition, and low VCAM-1 expression. PMA-treated samples demonstrated robust NET formation with markedly increased C3 deposition and VCAM-1 expression. PR3 antibody treatment alone induced complement C3 deposition without NET formation or increased VCAM-1 expression. PR3 combined with TNF-α triggered significant NET release, increased C3 deposition, and elevated VCAM-1 expression. Recombinant TSP-1 prevented NET formation, reduced C3 deposition, and normalized VCAM-1 expression in PR3- and TNF-α–treated samples. Blocking TSP-1 with an antibody (A6.1) in PR3-treated blood promoted NET formation, increased C3 deposition and VCAM-1 expression; effects were reversed by C5a receptor inhibition with avacopan. (**B**) Analysis of MFI of complement C3 deposition on HUVECs. (**C**) Analysis of MFI of VCAM-1 expression on HUVECs. (**D**) TSP-1 concentrations in supernatants were elevated with PMA and PR3 treatments. A6.1 treatment reduced detectable levels, while recombinant TSP-1 restored physiological levels. (**E**) Quantification of plasma C5a. C5a levels were significantly increased by PMA, TNF-α, and A6.1 treatment. Avacopan or recombinant TSP-1 reduced C5a concentrations. (**F**) Quantification of plasma histone DNA complexes. Increased amounts of DNA-histone complexes were detected in samples treated with PMA, TNF-α, and A6.1; treatment with recombinant TSP-1 or avacopan reduced DNA-histone complexes. Data are shown as mean ± SD of 3 independent experiments. *P ≤ 0.05, **P ≤ 0.01, ***P ≤ 0.001, NS, not significant; 1-way ANOVA was used with Tukey’s multiple-comparison test. Scale bar: 100 μm.

In untreated control samples, no NET formation was observed, and endothelial cells showed minimal C3 deposition and low VCAM-1 expression (Figure 7A). PMA stimulation induced robust NET formation, along with a marked increase in C3 deposition and VCAM-1 expression on endothelial cells (Figure 7, B and C). PR3 antibodies alone did not induce visible NET formation, consistent with the requirement for an additional inflammatory trigger in AAV. However, PR3 treatment significantly increased complement C3 deposition on endothelial cells without increasing VCAM-1 expression (Figure 7, B and C).

When TNF-α was added to PR3-treated blood, NET formation became clearly visible. VCAM-1 expression was significantly increased, indicating endothelial activation (Figure 7, A–C). In contrast, the addition of recombinant TSP-1 to TNF-α–treated samples prevented NET release, reduced C3 deposition, and normalized VCAM-1 expression, demonstrating a protective effect of TSP-1 in this model (Figure 7, A–C). Depleting TSP-1 in PR3-treated samples led to pronounced NET formation, comparable to TNF-α stimulation, with significantly increased C3 deposition and VCAM-1 expression. To determine whether complement drives TSP-1–mediated regulation of NETosis, avacopan, a C5a receptor inhibitor, was added alongside TSP-1 blockade. Avacopan effectively reduced C3 deposition, VCAM-1 expression, and NETosis, confirming that complement regulation by TSP-1 plays an important role in NET formation (Figure 7, A–C).

Supernatant measurements further underlined these findings. PMA-treated samples showed a substantial increase in TSP-1 levels (Figure 7D). Similarly, PR3 treatment also elevated TSP-1 levels, reflecting patient data. Blocking TSP-1 significantly reduced its detectable levels in plasma. C5a levels increased significantly after PMA stimulation, whereas no increase in C5a levels could be detected in samples treated with PR3 only (7E). However, when PR3 antibodies were used in conjunction with TSP-1 blockade, C5a levels increased significantly, correlating with the observed NET formation. In contrast, treatment of PR3-treated samples with TNF-α and recombinant TSP-1 significantly reduced C5a levels and suppressed NET formation (Figure 7E). Avacopan treatment effectively prevented NET formation in this experiment, but it did not significantly reduce C5a levels. This is expected, as avacopan is a C5a receptor antagonist and does not inhibit complement activation.

DNA-histone complex levels, indicative of NET release, were significantly elevated in samples treated with PMA; PR3 and TNF-α; and PR3 and A6.1 (Figure 7F). Addition of recombinant TSP-1 to TNF-α–treated samples significantly lowered DNA-histone complexes. Similarly, avacopan treatment reduced the level of DNA-histone complexes in samples treated with PR3 and A6.1. This indicates that the mechanism of NET formation caused by blocking TSP-1 in PR3-treated blood samples was complement-mediated. Taken together, these results demonstrated a clear regulatory role of TSP-1 in modulating complement activation and NETosis, important drivers of the pathogenesis in AAV.

### In vivo vasculitis model to investigate the role of TSP-1 in AAV.

**Figure 8:**
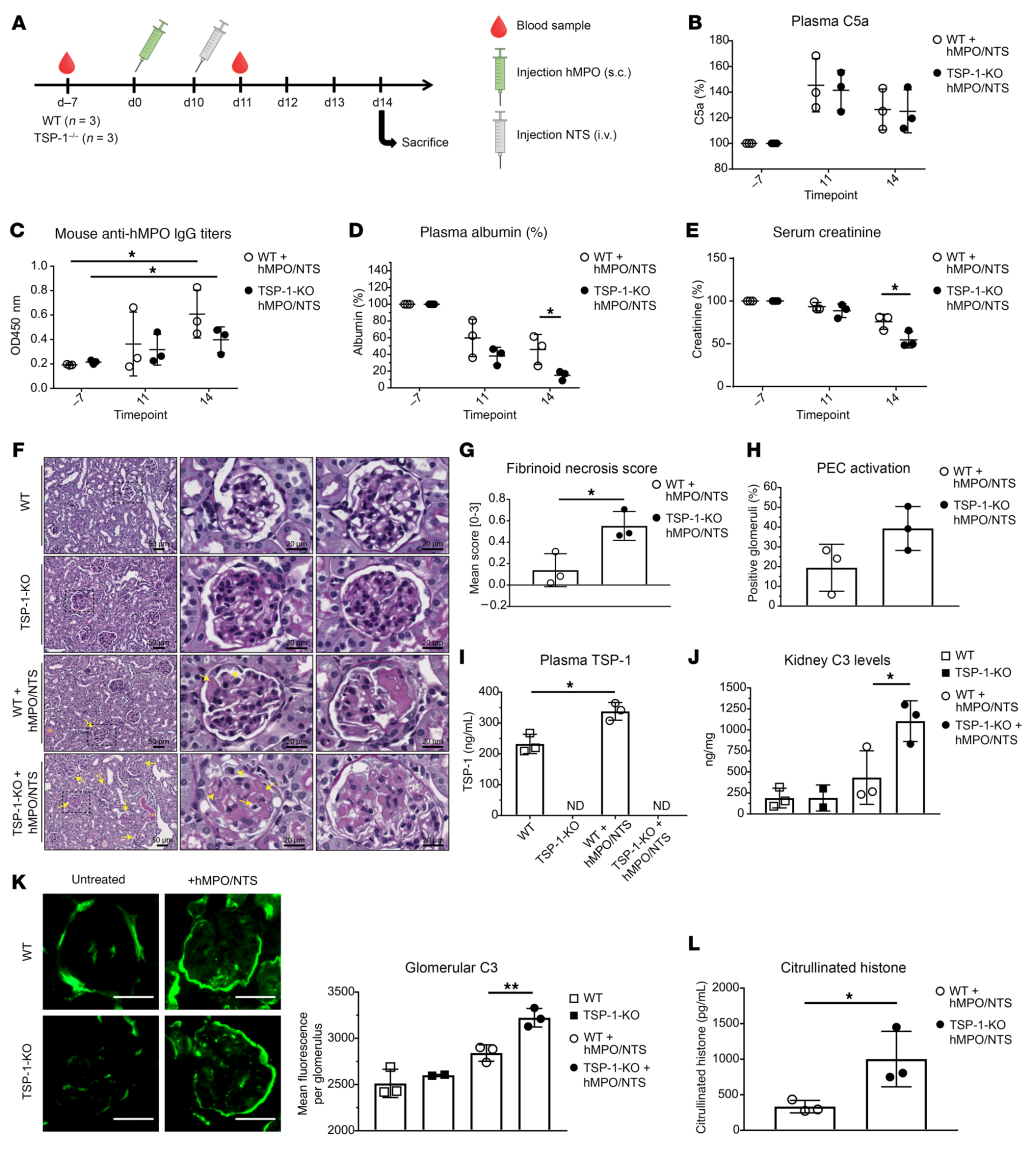
The lack of TSP-1 aggravates renal injury in a mouse model of AAV. (**A**) Experimental setup for murine model of AAV using TSP-1-KO and WT mice. On day 0, mice were immunized with hMPO and 10 days later injected with NTS. Blood was collected on day -7 and day 11; mice were euthanized on day 14. (**B**) Mice demonstrated significantly increased C5a levels and developed MPO antibodies (**C**), confirming disease induction. Data were normalized to day –7 (*n* = 3). (**D**) Plasma albumin levels showed more severe hypoalbuminemia in TSP-1-KO mice compared to WT mice at day 14. Data were normalized to day –7 (*n* = 3). (**E**) Serum creatinine levels were significantly reduced in TSP-1-KO mice compared with controls (*n* = 3). (**F**) Representative images of PAS-stained kidney sections from WT and TSP-1-KO mice. Mice developed fibrinoid necrosis and proteinaceous casts (asterisks). Glomeruli show thickening of capillary walls, parietal epithelial cell (PEC) activation (arrowheads), and capillary thrombi (arrows). (**G**) Quantification of glomeruli with fibrinoid necrosis shows significantly higher injury in TSP-1-KO mice compared with WT (*n* = 3). (**H**) TSP-1-KO mice trend toward greater PEC activation, precursor to crescent formation (*n* = 3). (**I**) Plasma TSP-1 levels were significantly increased in hMPO/NTS-treated WT mice, similar to AAV patients (*n* ≥ 2). (**J**) C3 levels in kidney lysates of hMPO/NTS-treated TSP-1-KO mice were significantly increased compared to WT mice (*n* ≥ 2). (**K**) Representative images and analysis of glomerular C3 deposition demonstrating significantly increased C3 deposits in hMPO/NTS-treated TSP-1-KO mice compared to WT mice (*n* ≥ 2). Scale bar: 40 μm. (**L**) Citrullinated histone levels were significantly higher in hMPO/NTS-treated TSP-1-KO mice, suggesting enhanced NET formation. Data are shown as mean ± SD. Statistical analysis by Student’s t test, *P < 0.05, **P < 0.01. ND, not detected.

To determine the functional significance of TSP-1 deficiency in AAV, we employed an established AAV mouse model (34). TSP-1-KO and WT mice were immunized subcutaneously with 10 μg of human myeloperoxidase (hMPO). Disease was initiated by intravenous injection of nephrotoxic serum (NTS) 10 days later. Mice were euthanized 4 days after NTS injection for analyses (Figure 8A).

Both TSP-1-KO and WT mice developed anti-MPO antibodies and exhibited elevated plasma C5a levels, confirming successful model induction (Figure 8, B and C). However, TSP-1-KO mice displayed a markedly more severe clinical phenotype compared with WT controls. TSP-1-KO mice developed ascites and weight gain, accompanied by significantly lower serum albumin levels, indicating an exacerbated nephrotic syndrome in the absence of TSP-1 (Figure 8D). Consistent with the distinct albumin loss, creatinine levels in the TSP-1-KO animals were significantly reduced, reflecting hyperfiltration typically observed in this early stage of the disease (Figure 8E).

Histopathological analysis of kidney sections stained with periodic acid–Schiff revealed significantly more glomeruli with fibrinoid necrosis in TSP-1-KO mice compared with WT mice (Figure 8, F and G). Although glomerular crescents were not yet evident at this early disease time point, TSP-1-KO mice showed a trend toward increased activation of parietal epithelial cells, which are known precursors of crescent formation (Figure 8, F and H). Furthermore, in accordance with elevated TSP-1 levels observed in patients with AAV and in our AAV microfluidic experiment, we also detected increased TSP-1 levels in WT mice after disease induction (Figure 8I).

Given that TSP-1 regulates the alternative complement pathway, we assessed complement C3 levels in the kidneys through ELISA of kidney tissue lysates and immunofluorescence staining of kidney sections. The hMPO/NTS model resulted in elevated C3 levels in the kidneys, confirming complement involvement in the disease (Figure 8J). In line with TSP-1’s role as an alternative pathway inhibitor, we observed a significant increase in complement C3 in tissue lysates from TSP-1-KO kidneys. Moreover, immunofluorescence staining showed significantly increased C3 deposition in the glomeruli of TSP-1-KO mice compared with WT controls (Figure 8K). To assess the impact of TSP-1 on NETosis, we measured citrullinated histone H3 levels in plasma as a marker of NET release. TSP-1-KO mice exhibited significantly higher plasma levels of citrullinated histone H3, indicating enhanced NET formation (Figure 8L).

## Discussion

**Figure 9:**
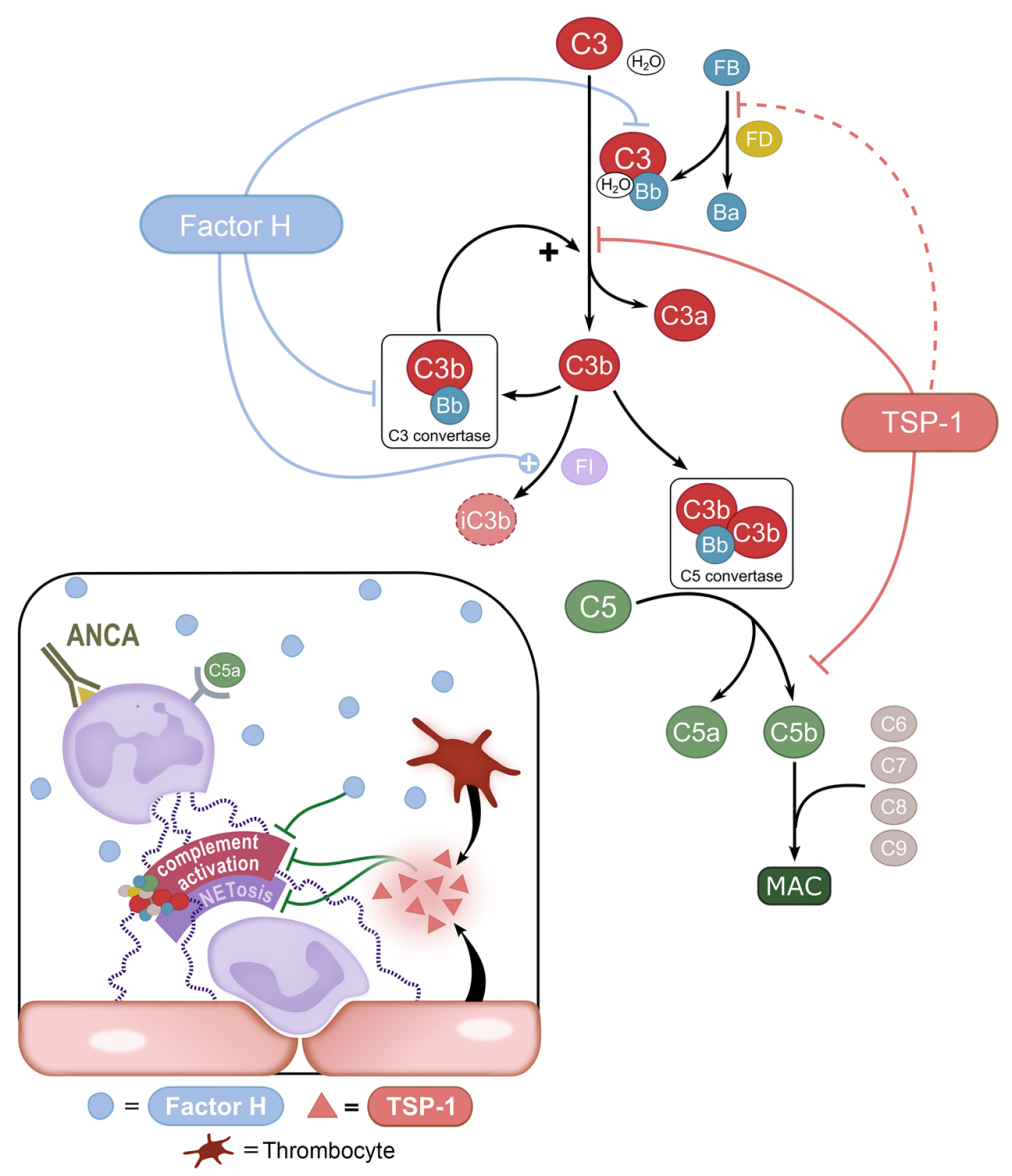
Summary of TSP-1 effects on the alternative pathway and physiological significance. Complement inhibitory functions of TSP-1 in comparison to FH are schematically illustrated: TSP-1 prevents cleavage of FB by FD in vitro, although binding to FB has not been confirmed by SPR and therefore not physiologically relevant (dashed line). TSP-1 binds to C3 and prevents the cleavage of C3 into C3a and C3b. Furthermore, TSP-1 binds to C5 and prevents its cleavage into C5a and C5b and the formation of MAC. Possible physiological and pathophysiological complement regulatory functions of TSP-1 in AAV: secondary complement activation in AAV due to neutrophil activation, NETosis, and subsequent vasculitis. In these local overwhelming conditions, inhibition by FH might not be sufficient to control complement activation. Therefore, additional complement inhibitory functions by locally released TSP-1 from endothelia and/or thrombocytes could be physiologically relevant regarding control of excessive complement activation, especially on surfaces.

Our results establish TSP-1 as a potentially novel inhibitor of alternative complement pathway activation. Through biochemical and functional analyses, we identified interaction partners of TSP-1 and characterized its inhibitory function at both C3 and C5 levels of the complement cascade. TSP-1 effectively prevents C3 and C5 cleavage and subsequent MAC formation, representing a unique mechanism of complement regulation (summarized in Figure 9).

To substantiate TSP-1–dependent complement regulation in a more physiological context, we used various sera and erythrocytes from patients with primary complement-mediated diseases in ex vivo assays. In aHUS sera, TSP-1 was able to prevent complement activation and subsequent hemolysis of sheep erythrocytes and inhibited the deposition of C3 on the surface of endothelial cells. On PNH erythrocytes, the addition of TSP-1 prevented C3 deposition (Supplemental Figure 4). Additionally, we successfully demonstrated complement inhibition and a reduction in renal complement deposition in an in vivo C3 glomerulopathy mouse model through the administration of recombinant TSP-1. All these experiments demonstrated that TSP-1 can influence and prevent complement activation under different pathological conditions in vitro and in vivo.

The complement regulatory role of TSP-1 certainly differs from that of FH, as evidenced by their distinct phenotypes in KO models. Unlike FH-KO mice, which develop primary complement-related disorders resembling C3 glomerulopathy (29), TSP-1-KO mice show no renal phenotype (35). This observation aligns with our analysis of kidney sections from patients with C3 glomerulopathy, where TSP-1 expression remained unchanged (Figure 6).

These distinct phenotypes reflect the different distribution and modes of action of both proteins: FH is abundantly present in the bloodstream and is able to control complement activation at initiation, especially on host surfaces (36). In contrast, TSP-1 is only found in small amounts in the fluid phase but can be rapidly released from activated endothelial cells and/or platelets (2, 3). In inflammatory conditions, TSP-1 might act locally as a complement regulator, either alongside or in combination with FH. It is noteworthy that the complement-regulating properties of TSP-1 are functionally distinct from those of FH (Figure 9), suggesting a collaborative role for these two complement inhibitors. Moreover, TSP-1 does not influence decay-acceleration activity of FH and has no intrinsic cofactor activity (Supplemental Figure 2, A–D).

According to our assessment, TSP-1 deficiency does not play a major role in primary complement diseases. However, its local complement inhibitory effect may be physiologically significant in diseases where secondary complement activation plays a role in the pathomechanism alongside endothelial activation. In our experiments, siRNA-mediated knockdown of TSP-1 on endothelial cells led to increased complement C3 deposits and endothelial cell activation, and addition of TSP-1 could partially remodify this phenotype. Based on our results, the local complement inhibitory effect of TSP-1 may have physiological relevance in complement-associated vasculitis diseases such as AAV (21, 37).

In AAV, complement activation and NETosis are prominent drivers of vascular inflammation and injury (22). Complement component C5a is capable of triggering NETosis, whereupon the formed NETs serve as a platform for complement activation. Under these circumstances, locally released TSP-1 may additionally help to prevent excessive complement activation in this detrimental cycle. Consistent with this, significantly elevated plasma TSP-1 levels have recently been demonstrated in patients with AAV (657.47 ± 64.17 pg/mL) compared with healthy controls (253.78 ± 37.10 pg/mL, *P* ≤ 0.005) (30, 31).

Correspondingly, we detected prominent TSP-1 staining in renal biopsies of patients with AAV in glomerular crescents. Antineutrophil cytoplasmic antibodies have been used previously in conjunction with TNF-α stimulation to cause NETosis in a microfluidic experimental setting (32, 38). By utilizing a similar experimental setup, we could show that TSP-1 plays a crucial role in regulating complement activation and NETosis. We demonstrated that addition of TSP-1 could inhibit NET release and conversely that blockade of TSP-1 with an antibody led to significant NETosis even without an additional secondary trigger like TNF-α. This effect is complement-mediated, as addition of recombinant TSP-1 simultaneously leads to a reduction in C3 deposition on surfaces and decreased C5a levels in the supernatant. Notably, the effect of TSP-1 depletion, affecting both NETosis and complement parameters, could be reversed by C5a blockade. This substantiates the influence of TSP-1 on NETosis through complement inhibition.

Furthermore, we confirmed our in vitro findings by using an in vivo AAV mouse model. TSP-1 deficiency in this model led to a significantly more severe nephrotic phenotype and histopathology compared with WT mice. In accordance with our previous results, complement C3 deposits and NETosis markers were significantly elevated in TSP-1-KO mice, further supporting the role of TSP-1 in complement regulation and subsequent NETosis in vasculitis. Recently, Lucientes-Continente et al. utilized a similar animal model to the one used in our study in conjunction with mice heterozygous for FH deficiency (*FH^+/–^*) (39). *FH^+/–^* mice exhibited a more severe histomorphological phenotype as well as enhanced glomerular complement deposition compared with WT mice, similar to our observations in TSP-1-KO mice. These results highlight the pathophysiological role of complement in this disease and further indicate a collaborative role between FH and TSP-1 as complement inhibitors in AAV.

In this study, we demonstrated the complement inhibitory function of TSP-1 in human blood samples from healthy individuals and from patients with complement-related diseases, as well as in serum-free conditions with various assays. We initially used heparin-free TSP-1 with an N-terminal histidine tag, which could be a source of error, since TSP-1 can bind to histidine-rich proteins, potentially causing unspecific aggregation (40). To address this issue, we used untagged TSP-1 purified from human platelets and repeated our main findings (Figure 1B and Supplemental Figure 1). The complement regulatory functions of platelet-isolated TSP-1 remained consistent in our assays, excluding unspecific protein aggregation as the cause of complement regulation.

Although TSP-1 inhibits FD-mediated cleavage of FB in vitro, SPR measurement detected only weak binding to FB, suggesting that FB cleavage might not be essential under physiological conditions. In contrast, strong binding in SPR was confirmed for C3, C5, C8, and FH, suggesting that the TSP-1 effects on C3 and C5 cleavage and MAC formation are physiologically relevant processes.

TSP-1 is known to have various physiological effects stemming from interactions with numerous receptors and other proteins (41, 42). Therefore, in the more complex in vivo setting, complement regulation by TSP-1 may be additionally influenced by other effector pathways of TSP-1. However, by employing different model systems of various complement-mediated diseases, we were able to show a consistent inhibitory effect of TSP-1 on the alternative complement pathway. Our data clearly demonstrated TSP-1’s role in complement regulation, and it was crucial to address potential alternative mechanisms, given TSP-1’s well-known functions in other pathways. TSP-1 is particularly recognized for its profibrotic and antiangiogenic roles through the activation of TGF-β and CD47 signaling pathways, respectively (8, 9). However, our findings in AAV argue against these pathways as primary mediators of the observed effects. Previous studies have shown that TGF-β stimulation increases NET formation in neutrophils from patients with oral lichen planus, characterized by elevated levels of myeloperoxidase, citrullinated histone H3, and cell-free DNA (43). Similarly, CD47 blockade has been found to reduce NET release and ameliorate renal injury in a mouse model of crescentic glomerulonephritis (44). If these pathways were the predominant mechanism of TSP-1’s action in our model, TSP-1 deficiency should have resulted in reduced NET formation and a protective phenotype. Instead, we observed the opposite: TSP-1–deficient mice showed a more severe disease phenotype. These findings strongly support our conclusion that TSP-1’s complement regulatory function, rather than its effects through TGF-β or CD47, is the primary mechanism in AAV. Although TSP-1 mutations have been reported in pulmonary arterial hypertension and congenital glaucoma (45, 46), reduced functional TSP-1 has not yet been investigated in patients with AAV. However, complete loss-of-function mutations appear to be incompatible with human development (29, 41).

Overall, our findings provide strong evidence that TSP-1 acts as a potentially novel complement inhibitor. Although complement regulation by TSP-1 appears less relevant under physiological conditions, it seems to be an important co-player in the context of pronounced endothelial inflammation. Hence, it represents a potentially new mechanism in the intricate interplay between endothelia and complement. The regulatory effect in particular gains importance in secondary complement-mediated vasculitis disease, where additional complement inhibitory function is required locally because of excessive complement-activating processes (Figure 9). Given its inhibitory effect, particularly on surface complement activation, and its interaction with vWF, it is intriguing to hypothesize that TSP-1 protects endothelial cells from complement-mediated damage.

Considering that complement activation itself can contribute to vasculitis or thrombotic conditions, TSP-1 may also be involved in mitigating the sequelae of an overactive complement system, further preventing anaphylatoxin C3a and C5a formation (18, 19, 47). Therefore, our findings may contribute to an improved understanding of these complex pathogenic mechanisms and might pave the way for the development of potentially novel therapeutic strategies.

## Methods

### Sex as biological variable.

Sex was not considered as a biological variable with the exception of AAV in vivo experiments. Blood and tissue samples from patients examined in our study were from both sexes. Patient details are summarized in Supplemental Tables 1–3. In experiments involving FH-deficient mice, male and female mice were used for experiments. In experiments using WT and TSP-1-KO mice, only male mice were used because female mice were more resistant against the AAV model and developed fewer renal lesions.

### Generation and purification of TSP-1.

Human TSP-1 cDNA was obtained from Sinobiological (HG10508-UT) and subcloned into a pFastbac1 vector containing a gp67 signal peptide for secretion and an N-terminal 10xHis tag for purification. Bacmids were generated using the Bac-to-Bac vector kit (Thermo Fisher Scientific, 10360014) and used to generate recombinant baculovirus. TSP-1 was expressed in Spodoptera frugiperda 9 (Thermo Fisher Scientific, 11496015) cells and purified from cell supernatants via Ni-NTA columns. TSP-1 concentrations were determined by ELISA (R&D Systems, DY3074).

### Proteins and sera.

Complement proteins (FB, FD, FH, FI, C3, C3b, C5, C5b6, C6, C7, C8, and C9) as well as FH-deficient serum were obtained from Complement Technologies. CVF was purchased from Quidel, and eculizumab (Soliris) was obtained from remnants of infusions. TSP-5 was obtained from R&D Systems (3134-CPB). Serum samples from healthy donors or patients with aHUS and EDTA-treated whole blood from patients with PNH were obtained following standard procedures (48). PR3 antibodies from a patient with AAV1 were obtained by purifying plasma using a protein G affinity column (Cytiva) following the manufacturer’s instructions. PR3 concentrations were determined by the rheumatology-immunology laboratory of the University Medical Center Freiburg using a fully automated ELISA (Alegria).

### Alternative pathway ELISA.

Alternative pathway activity was measured using commercially available ELISA kits following the manufacturer’s instructions (Avar Life Science, COMPLAP330). C5a was quantified by C5a ELISA (Abcam, ab193695) according to the manufacturer’s protocol with the following modification: during the incubation period, 30 μL of ice-cold 20 mM EDTA was added to the samples. Cell death detection ELISA (Roche, 11544675001) was used to detect histones H1, H2a, H2b, H3, and H4 single- and double-stranded DNA from microfluidic experiments according to the manufacturer’s instructions.

### Hemolytic assay.

Sheep erythrocytes (Fiebig Nährstofftechnik) were washed and diluted with GVB buffer containing MgEGTA, and the experiment was performed as described before (49). Released hemoglobin from erythrocytes was measured via absorption measurement at 414 nm.

### TSP-1 binding ELISA to complement proteins.

Complement proteins FH, FB, C3, C5, C6, C7, C8, C9, or BSA were coated on microtiter plates at a concentration of 133 nM each in PBS overnight at 4°C on Nunc Maxisorb 96-well plates. Unbound proteins were washed with PBS containing 0.05% Tween 20 and the wells blocked with PBS containing 2% BSA for 1 hour at room temperature. After washing, recombinant TSP-1 was added to the wells at 10 μg/mL and incubated for 2 hours at room temperature. Bound TSP-1 was detected using a mouse anti-human TSP-1 antibody (Merck, MABT879) for 2 hours at room temperature, followed by a sheep anti-mouse HRP secondary antibody (GE Healthcare, NA931V) for 1 hour at room temperature. Colorimetric detection was performed using TMB substrate. The reaction was stopped after 10 minutes, and OD at 450 nm was measured.

### SPR Biacore measurements.

Kinetic analysis of the interaction between TSP-1 and complement proteins was performed on a BIAcore 3000 instrument (Biacore AB). TSP-1 was bound to CM5 chips at a concentration of 0.1 μM via covalent bond. Binding to complement proteins FH, FB, C3, C3b, C5, and C8 was assessed over a range of concentrations (12.3, 37.03, 111.1, 333.3, 1,000 nM) in PBS plus 0.05% Tween 20. Binding data were fitted to a 1:1 Langmuir binding model, and on and off rates were determined for calculating affinity constants. Between experiments, the chip surface was regenerated with 10 mM sodium hydroxide.

### FB cleavage assay.

The ability of TSP-1 to inhibit FD-mediated cleavage of FB was performed as described before (50). FB cleavage products were visualized with Coomassie blue staining after SDS-PAGE. Uncleaved and cleaved FB band intensities were determined using ImageJ (NIH).

### C3 convertase assay.

C3 convertase activity was assessed as described before (50). Briefly, C3(H_2_O) was generated by incubating C3 with 200 mM methylamine for 30 minutes at 37°C. The convertase was generated by incubating C3(H_2_O) with FB and FD. As a positive control, CVF was incubated with FB and FD instead of C3(H_2_O). After stopping the reaction, increasing amounts of TSP-1 and C3 were added to the mixture, and the amount of generated C3-α′ was visualized with a Coomassie blue stain after SDS-PAGE. C3-α′ band intensity was determined using ImageJ.

### C5 convertase assay.

First, 25 nM CVF, 25 nM FB, and 2.5 nM FD were diluted in PBS containing 5 mM MgCl_2_. The reaction was incubated for 1 hour at 37°C to build up C5 convertase (CVFBb). Next, 200 nM of C5 was mixed with a 16-fold molar excess of test proteins and incubated for 30 minutes at room temperature. After incubation, the 2 samples were mixed, and the amount of released C5a was quantified by ELISA (R&D Systems, DY2037).

### MAC formation assay.

The ability of TSP-1 to inhibit MAC formation on the surface of sheep erythrocytes was performed as described before (49). The amount of released hemoglobin was determined by OD measurement of supernatants at 414 nm.

### Inhibition of C3 deposition on HMEC-1.

The ability of TSP-1 to inhibit C3 deposition on endothelial cells was assessed with an assay described previously (26). Briefly, HMEC-1 cells were activated with 10 μM ADP in HBSS for 10 minutes. Next, cells were incubated with either normal human serum or aHUS patient serum treated with or without 1 μM of FH or TSP-1 for 4 hours at 37°C. After incubation the cells were fixed in 4% PFA and stained with rabbit anti-human C3c FITC antibody (1:100, Dako, F020102), capable of recognizing C3 and C3b, and DAPI. The mean fluorescence of obtained images was measured using ImageJ.

### siRNA-mediated knockdown of TSP-1.

siRNA to knock down TSP-1 expression in HUVECs was obtained from QIAGEN (GS7057). AllStars siRNA (QIAGEN) was used as a negative control. Transfection was performed using Oligofectamine 2000 (Invitrogen) reagent in Gibco 199 serum-free medium following the manufacturer’s instructions. The cells were incubated for 48 hours at 37°C before use in experiments.

On the day of the experiment, HUVECs were treated with HEPES buffer containing 50 μM histamine with or without TSP-1. The cells were incubated at 37°C for 45 minutes, washed with HEPES, and fixed with 4% PFA. The cells were blocked with 1% BSA before staining. HUVECs were stained with goat anti-human C3 (1:100, Complement Technologies, A213) and mouse anti-human TSP-1 (1:100, Santa Cruz Biotechnology, sc-59887), followed by donkey anti-mouse Alexa 488 (1:500) and donkey anti-goat IgG Alexa 555 (1:500, Thermo Fisher Scientific, A21432). Nuclei were stained with DAPI before mounting with Mowiol. The mean fluorescence of obtained images was measured using ImageJ. In parallel experiments, transcriptional regulation of VCAM1, C3, CD55, CD59, and CD46 in treated HUVECs was analyzed by qPCR. RNA was purified using TRIzol reagent and cDNA generated by GoScript Reverse Transcriptase according to the manufacturer’s instructions (Promega, A5001). qPCR reactions were performed using GoTaq qPCR master mix (Promega, A6001). Primers used for this study are summarized in Supplemental Table 4. Data were normalized to the expression of the housekeeping gene β-actin or GAPDH using the 2-ΔΔct method.

### Staining TSP-1 in renal biopsies.

IHC staining of FFPE kidney biopsy and cancer nephrectomy samples was performed by applying standard procedures as previously described (51). After heat-induced antigen retrieval (citrate buffer, pH 6) of samples, an anti-TSP1 antibody (1:400, Merck MABT879) was diluted in blocking solution and incubated at 4°C overnight. Samples were washed in PBS and HRP-linked secondary antibody (1:500, Dako, P0447) was diluted and applied for 30 minutes. Finally, samples were washed in PBS. DAB chromogen (Dako, K3468) staining reagent was applied following the manufacturer’s instructions and counterstained using hematoxylin. Samples were digitalized using a slide scanner (Ventana DP 200, Roche Diagnostics).

### In vitro AAV model.

A microfluidic system with an air pressure–based pump system (Ibidi GmbH) was used. HUVECs were first cultured on gelatin-coated 0.2 Luer μ-slides (Ibidi GmbH) for 48 hours prior to the experiment. For microfluidic experiments, approximately 350,000 cells per slide were used.

On the day of the experiment, HUVECs were washed once with HEPES and consecutively perfused (pressure: 16.2 mbar, shear rate: 5 dyn/cm², flow rate: 0.5 mL/min) with hirudinized whole blood (1:1 mixed with HEPES). Prior to perfusion, hirudinized blood was treated as follows: untreated, PR3 (250 U/mL final concentration);PR3 with TNF-α (20 ng/mL final concentration); PR3 with TNF-α and recombinant TSP-1 (400 nM final concentration); PR3 with TSP-1 antibody (A6.1, 5 μg/mL final concentration, Santa Cruz Biotechnology, sc-59887); and PR3 with A6.1 and avacopan (400 ng/mL). Additionally, DAPI was added to whole blood as a marker for live imaging of DNA NET release. Untreated hirudinized whole blood was used as negative control. After 4 hours of incubation, supernatants were centrifuged and collected for analysis of TSP-1, C5a, and released histones and DNA. HUVECs were fixed and stained for C3 (1:200, Complement Technologies, A213) and VCAM-1 (1:400, Thermo Fisher Scientific, MA5-31965) for immunofluorescence imaging.

### Animal experiments.

All procedures involving animals were conducted in accordance with the *Guide for the Care and Use of Laboratory Animals* (National Academies Press, 2011) and the German Animal Protection Code and were approved by local authorities (Regierungspräsidium Freiburg, G21/111, G24/064, G24/086).

FH-depleted mice were provided by Matthew Pickering (Centre for Complement and Inflammation Research, Imperial College London, London, United Kingdom). Male and female mice 8–16 weeks of age were used for the experiments. On the day of the experiment, the mice were injected intravenously with 400 μg TSP-1 or PBS. Blood samples were collected immediately before injection (0 hour) and at subsequent time points (0.5, 1, 2, 4, 6, and 24 hours). Blood was collected into EDTA-coated capillaries and centrifuged at 2000*g* for 10 minutes to obtain plasma for analysis. Mice were euthanized 24 hours after injection for blood and kidney collection.

For AAV experiments, 8- to 12-week-old male TSP-1-KO (B6.129S2-Thbs1tm1Hyn/J) and age-matched male WT control mice were obtained from the Jackson Laboratory. On day 0, mice were immunized subcutaneously with 10 μg of hMPO (R&D Systems, 3174-MP-250) emulsified in TiterMax Gold adjuvant (Sigma-Aldrich, T2684). Ten days later, NTS (Probetex, PTX-001S) was administered intravenously following a previously described protocol (34). Blood samples were collected from the tail vein 1 day after NTS injection (day 11). On day 14, mice were injected with an intraperitoneal injection of ketamine (100 mg/kg) and xylazine (15 mg/kg) and euthanized by exsanguination. Kidneys were snap-frozen in liquid nitrogen or fixed in 4 % PFA for histological and functional analyses.

### ELISA.

The following ELISAs and sample dilutions were used in this study: mouse albumin ELISA kit (1:500.000, Crystal Chem, 80630), mouse creatinine (Cr) ELISA kit (1:10, AFG Scientific, EK734005), mouse complement C3 ELISA kit (1:300, Abcam, AB157711), mouse Cit H3 ELISA kit (1:10, Biorbyt, ORB1496437), mouse C5a ELISA kit (1:25, Antikoerper Online, ABIN6720057), and mouse TSP-1 (THBS1) ELISA kit (1:60, Biozol, ABX575980). Concentrations were determined according to the manufacturers’ instructions.

### Determination of α-hMPO antibodies.

Recombinant hMPO (R&D Systems, 3174-MP-250) was coated onto 96-well plates in coating buffer (15 mM Na_2_CO_3_, 35 mM NaHCO_3_, pH 9.6) at a concentration of 5 μg/mL overnight at 4°C. The wells were washed 3 times with washing buffer (PBS plus 0.05% Tween 20) and blocked for 3 hours at 37°C in blocking buffer (PBS plus 3% BSA). After blocking, the wells were washed 3 times and incubated with plasma samples diluted 1:400 in dilution buffer (PBS plus 1% BSA) for 1 hour at 37°C. The wells were washed 4 times and incubated with a sheep anti-mouse IgG HRP antibody (GE Healthcare, NXA931) diluted 1:10,000 in washing buffer for 30 minutes at 37°C. The plate was washed 4 times and incubated with TMB substrate (Thermo Fisher Scientific, N301) for 15 minutes. The reaction was stopped with H_2_SO_4_, and the absorbance at 450 nm was measured.

### Generation of kidney lysates.

Snap-frozen kidneys (30–50 mg) were placed in a 2 mL tube containing 1.4 mm zirconium beads (Bertin Technologies, P000912-LYSK0-A) and lysis buffer (140 mM NaCl, 1% Triton X-100, 50 mM Tris pH 7.4) supplemented with proteinase inhibitors without EDTA (Thermo Fisher Scientific, 87786). The samples were homogenized using a Minilys Personal Homogenizer (Bertin Technologies) for two 30-second intervals at 5500 rpm, with a 30-second pause between intervals. The resulting suspension was transferred to a new tube and centrifuged for 10 minutes at 20,000*g* at 4°C to remove cell debris. The clear lysate was aliquoted and immediately stored at –80°C until further analysis.

### Periodic acid–Schiff staining of renal mouse tissues.

FFPE of 2 μm thick kidney sections and periodic acid–Schiff staining were performed as previously described (51). The presence of glomerular fibrinoid necrosis and associated thrombotic occlusion of glomerular capillaries, as well as parietal epithelial cell activation (defined as proliferation, protrusion into the Bowman’s space, and thickening of the Bowman’s capsule) was assessed in 97 to 108 glomeruli per animal. A semiquantitative score was employed to quantify the extent of fibrinoid necrosis within the glomerular tuft (0, no fibrinoid necrosis; 1 less than 25%; 2, 25%–50%; and 3, more than 50% of the tuft area).

### Staining of C3 in renal mouse tissues.

First, 5 μm cryosections from mouse kidneys were fixed in 4% paraformaldehyde solution and permeabilized in PBS containing 0.5% Tween 20. C3 was detected by incubating the slides with a directly labeled goat anti-mouse C3 FITC antibody (1:400, MP Biomedicals, 0855500) overnight at 4°C in a wet chamber. After washing, slides were mounted using Fluoromount-G with DAPI (Thermo Fisher Scientific, 00-4959-52). For quantitative immunofluorescence staining, MFI of at least 30 glomeruli per animal was measured using ImageJ.

### Statistics.

All graphs and statistics were created using GraphPad Prism version 8. One-way ANOVA with post hoc Tukey’s multiple-comparison test or 2-tailed Student’s *t* test was used for statistical evaluation as indicated. *P* values of less than 0.05 were considered significant.

### Study approval.

This study is registered at the German clinical trials register (DRKS00025182) and was approved by the ethics committee of Freiburg (No. 21-1324 and 21-1288). All patients included in this study provided written consent. Patients’ details are provided in Supplemental Tables 1–3.

### Data availability.

Values for all data points in graphs are reported in the Supporting Data Values file. For original data, please email the corresponding author.

## Author contributions

SK, BZ, TT, and KH designed the experiments. SK, JK, XL, and TT performed the research and collected the data. SK, HW, and RG performed SPR analysis. MR and CS performed kidney biopsy staining for TSP-1 and histological analysis of mice samples. SV, XL, and CG performed microfluidic experiments. SK, SS, CG, TT, and KH analyzed the data. MP, JP, and KH provided the clinical data. SK, SS, ELD, MP, BZ, JP, TT, and KH wrote the manuscript, which was revised by all other authors.

## Supplementary Material

Supplemental data

Unedited blot and gel images

Supporting data values

## Version 1 05/08/2025

In-Press Preview

## Version 2 07/01/2025

Electronic publication
