# Supplementary material for: Thrombospondin-1 inhibits alternative complement pathway activation in antineutrophil cytoplasmic antibody-associated vasculitis
Source: J Clin Invest. 2025 May 8;135(13):e180062. doi: 10.1172/JCI180062 (PMC12208556; doi:10.1172/JCI180062)
Supplement: Supplemental data [file jci-135-180062-s112.pdf]

# Thrombospondin-1 inhibits alternative complement pathway activation in antineutrophil cytoplasmic antibody-associated vasculitis

## Supplemental

### Patient Details

Blood samples were collected from aHUS patients/relatives, PNH, ANCA-associated vasculitis patients and controls. Patients' details are described in supplemental table 1-3.

## Supplemental Tables

### Supplemental Table 1

### aHUS

| | Age | Gender | Genetics | Autoantibodies | Creatinine (mg/dl) | Serum C3 (g/l) | Proteinuria (g/g creatinine) | Actual complement therapy |
|---|---|---|---|---|---|---|---|---|
| **aHUS1** | 34 | Male | *het. FH-* mutation R1215Q | No | 0,89 (0,6-1,2) | 1,1 (0,9-1,8) | 0,04 (<0,2) | No |
| **aHUS2** | 12 | Male | *homoz. CFHR1/3 deletion* | Anti-FH: 147U/ml (<10) | 2,46 (0,3 - 0,5) | 0,63 (0,9-1,8) | 3,03 (<0,2) | Ravulizumab[A]/ mycophenolate mofetil |
| **aHUS3** | 24 | Male | *het. FH-c-terminal deletion* | No | 2,64 (0,6 - 1,2) | <0,19 (0,9-1,8) | 0,24 (<0,2) | Ravulizumab[A] |

**Table S1:** Summary of clinical data of aHUS patients/relatives: aHUS1 is a healthy man from an aHUS family carrying a previously described *FH* mutation. The *FH* mutation causes strong hemolytic activity when serum is incubated with sheep erythrocytes. Het. is heterozygous, homoz. is homozygous mutation, A - Serum samples used for in vitro assays were taken before the initiation of complement therapy

## Supplemental Table 2

### PNH

| | Age | Gender | C59% negative erythrocytes (%) | Complement therapy |
|---|---|---|---|---|
| **PHN1** | 64 | Male | 99,5 | Iptacopan |
| **PHN2** | 77 | Female | *99,7* | Iptacopan |
| **PNH3** | 24 | Female | *47,9* | Pegcetacoplan |

**Table S2:** Summary of clinical data of PNH patients

## Supplemental Table 3

### ANCA-associated vasculitis/controls

| | Age | Gender | Histology (crescent/sclerosis of total glomeruli) | Autoantibodies[A] | Creatinine[A] (mg/dl) | Proteinuria[A] (g/g creatinine) |
|---|---|---|---|---|---|---|
| **Patient diagnosis & number** | | | | | | |
| **Nephrektomy1 (Oncocytoma)** | 71 | Male | Normal (0/0 of 15) | n.d. | 1,3 (0,6-1,2) | 0 |
| **Nephrektomy2 (Clear Cell Renal Cell Carcinoma)** | 68 | Male | Normal (0/0 of 50) | n.d. | 4,3 (0,6-1,2) | n.d. |
| **Nephrektomy3 (Clear Cell Renal Cell Carcinoma)** | 64 | Male | Normal (0/0 of 50) | n.d. | 2,1 (0,6-1,2) | 0,06 (<0,2) |
| **Nephrektomy4 (Clear Cell Renal Cell Carcinoma)** | 55 | Male | Normal (0/0 of 50) | n.d. | 1,1 (0,6-1,2) | n.d. |
| **AAV1** | 44 | Female | pauci-immune (21/3 of 34) | Anti-PR3: >200U/ml (<10) | 3,1 (0,5-1,0) | 1,18 (<0,2) |
| **AAV2** | 21 | Female | pauci-immune (5/3 of 13) | Anti-MPO: >200U/ml (<20) | 1,2 (0,5-0,8) | 0,5 (<0,2) |
| **AAV3** | 60 | Male | pauci-immune (7/3 of 12) | Anti-PR3: >200U/ml (<10) | 2,8 (0,6-1,2) | 2,94 (<0,2) |
| **AAV4** | 63 | Male | pauci-immune (1/0 of 6) | Anti-MPO: 120U/ml (<20) | 5,8 (0,6-1,2) | 1,25 (<0,2) |
| **FSGS1 Transplant; FSGS relapse** | 15 | Female | FSGS (0/7 of 50) | n.d. | 0,94 (0,5-1,0) | 6,52 (<0,2) |
| **FSGS2** | 65 | Male | FSGS (0/11 of 15) | n.d. | 2,76 (0,6-1,2) | 1,50 (<0,2) |
| **FSGS3** | 54 | Male | FSGS (0/4 of 9) | n.d. | 3,25 (0,6-1,2) | 2,49 (<0,2) |
| **FSGS4** | 19 | Female | FSGS (0/50 of 50) | n.d. | 4,1 (0,5-1,0) | 7,15 (<0,2) |
| **C3 Glomerulopathy1** | 22 | Female | MPGN/C3 glomerulopathy (0/5 of 18) | n.d. | 0,96 (0,5-0,9) | 0,1 (<0,2) |
| **C3 Glomerulopathy2** | 64 | Male | MPGN/C3 glomerulopathy (0/5 of 14) | n.d. | 3,5 (0,7-1,2) | 7,5 (<0,2) |
| **Diabetic Nephropathy1** | 82 | Female | Nodular glomerulosclerosis (0/2 of 10) | negative | 1,36 (0,5-0,9) | 3,9 (<0,2) |
| **Diabetic Nephropathy1** | 74 | Male | Nodular glomerulosclerosis (0/10 of 15) | negative | 3,4 (0,7-1,2) | 2,8 (<0,2) |

**Table S3:** Summary of clinical data of ANCA-associated vasculitis patients (AAV), controls (nephrectomy, FSGS (focal segmental glomerular sclerosis), C3 glomerulopathy and diabetic nephropathy. A - values at time of renal biopsy, n.d.: not determined

**Supplemental Table 4:**

| **Primer** | **Sequence** | **Amplicon Size** | **Reference** |
|---|---|---|---|
| THBS1 fw | TGCCTGATGACAAGTTCCAAG | 133 bp | (1) |
| THBS1 rv | CCAGAGTGGTCTTTCCGCTC | | |
| | | | |
| CD46 fw | TTGTGATCCTGCACCTGGAC | 102 bp | (2) |
| CD46 rv | TTACACTCTGGAGCAGCACG | | |
| | | | |
| CD55 fw | CCAAATGCTCAAGCAACACG | 128 bp | (2) |
| CD55 rv | AAACACGTGTGCCCAGATAG | | |
| | | | |
| CD59 fw | CTGCTGCAAGAAGGACCTGT | 105 bp | (2) |
| CD59 rv | GCTGCCAGAAATGGAGTCAC | | |
| | | | |
| GAPDH fw | ACAACTTTGGTATCGTGGAAGG | 101 bp | (3) |
| GAPDH rv | GCCATCACGCCACAGTTTC | | |
| | | | |
| bAct fw | CGACAGGATGCAGAAGGAG | 137 bp | (3) |
| bAct rv | ACATCTGCTGGAAGGTGGA | | |
| | | | |
| VCAM-1 fw | GTCAATGTTGCCCCCAGAGA | 128 bp | (4) |
| VCAM-1 rv | TGCCTGCTCCACAGGATTTT | | |
| | | | |

**Table S4:** Summary of qPCR primers used in the study. fw – forward, rv – Reverse

## Supplemental Methods:

### ELISA confirming binding to complement proteins with platelet isolated TSP-1 (p-TSP-1)

Binding of TSP-1 to complement proteins was confirmed using untagged TSP-1 isolated from human platelets. Recombinant TSP-1 (TSP-1), platelet-isolated TSP-1 (p-TSP-1), Eculizumab, BSA or, where applicable, FH was coated overnight on Nunc maxisorb 96 well plates at 133 nM each in PBS. Unbound proteins were washed with PBS containing 0.05 % Tween20 and the wells blocked with PBS containing 2 % BSA for 1 h at room temperature. After washing, human FH, FB, C3 or C5 was added to the wells at 10 or 20 µg/ml and incubated for 2 h at room temperature. After washing, complement proteins were detected by incubating the samples for 2 h at room temperature with the following specific primary antibodies: goat anti-human FH (Complement Technology, A237), goat anti-human FB (Calbiochem, 341272), goat anti-human C3 (Complement Technology, A213), mouse anti-human C5 (Quidel, A306). After washing, the samples were incubated for 1 h at room temperature with rabbit anti-goat HRP (Dako, P0449) or sheep anti-mouse HRP (GE-Healthcare, NXA931) secondary antibodies. Colorimetric detection of HRP antibodies was performed using TMB substrate. The reaction was stopped after 10 min and optical density at 450 nm measured.

### Cofactor and decay acceleration assay:

Cofactor activity of FH in the presence or absence of TSP-1 was assessed as described before (5). C3 cleavage products were visualized using coomassie staining after SDS-PAGE.

Decay acceleration activity of FH in the presence or absence of TSP-1 was measured by ELISA as previously described (6).

### Prediction of protein interaction by AlphaFold 3:

The prediction of the interaction of Thrombospondin-1 (UniProt P07996) with Complement factor H (UniProt P08603), Complement C3 (UniProt P01024) or Complement C5 (UniProt

P01031) was performed with AlphaFold 3 model (7) . The binding interface was identified upon the presence of hydrogen bonds shorter than 3.5 Å. The hydrogen bond identification and the structure editing were conducted using Pymol.

**Inhibition of PNH C3 deposition:**

The ability of TSP-1 to prevent C3 deposition on the surface of PNH erythrocytes was performed as described before (8). Briefly, EDTA blood from three different PNH patients (patient details in supplement Table S2) was obtained and washed three times in saline. 2 µl of erythrocytes were mixed with PBS alone or 0.5 µM Eculizumab with or without 1.5 µM TSP-1 or 12 µM Pegcetacoplan in a total volume of 10 µl. 30 µl of AB0 matched, acid activated (0.1 M HCl, 1:20 diluted) normal human serum (aNHS) supplemented with 2mM $MgCl_2$ was added to the cells and incubated for 24 h at 37 °C. The cells were washed with PBS containing 2 mM EDTA and stained with antibodies against CD59 (1:100, anti-human CD59 PE, Biolegend) and C3c (1:25, anti-human C3c FITC, Dako F020102) before flow cytometry analysis.

**Comparison of TSP-1 expression in HMEC-1 and HUVEC:**

Human umbilical vein endothelial cells (HUVEC, C-12203, Sigma) and human microvascular endothelial cells (HMEC-1, CRL-3243, ATCC) were cultured in T75 flasks. HUVEC were maintained in endothelial cell growth medium (Promocell, C-22010), while HMEC-1 were cultured in MCDB131 medium (ThermoFisher, 10372019). Once the cells reached approximately 80% confluency, equal amounts of culture supernatants were collected, and the cells were washed with PBS. Cells were then detached using TrypLE (ThermoFisher, 12604021), and cell counts were determined using a Neubauer chamber. A total of $1x10^6$ cells were harvested and lysed in 100 µl of cell lysis buffer (140 mM NaCl, 1 % TritonX-100, 50 mM Tris pH7.4) for 30 minutes on ice. The resulting cell suspension was centrifuged at 20,000 × g for 10 minutes, and the supernatant collected for further analysis. Protein concentrations of cell lysates were determined using a BCA protein assay (A55864). 10 µg of cell lysates were subjected to SDS-PAGE using a Mini-PROTEAN Tetra cell (Bio-Rad). After electrophoresis,

the proteins were transferred to a PVDF membrane (Hybond P, GE # 10600023). The membrane was washed in TBST buffer and blocked for 1 h at room temperature in blocking buffer (3 % BSA in TBST). The membrane was then incubated overnight at 4 °C with primary antibodies (TSP-1 A6.1, sc-59887, Santa Cruz 1:500). Beta-actin was used as a loading control in cell lysates (Beta Actin HRP, HRP-66009, Proteintech, 1:10000). After washing with PBS-T the membrane was incubated an anti-mouse HRP-conjugated secondary antibody (sheep anti mouse 1:2000). After washing, the membranes were treated with ECL substrate for visualization and bands were detected on an Azure280.

## Supplementary Figures

**Supplemental Figure 1: ELISA confirming BLI binding to complement proteins with platelet isolated TSP-1**

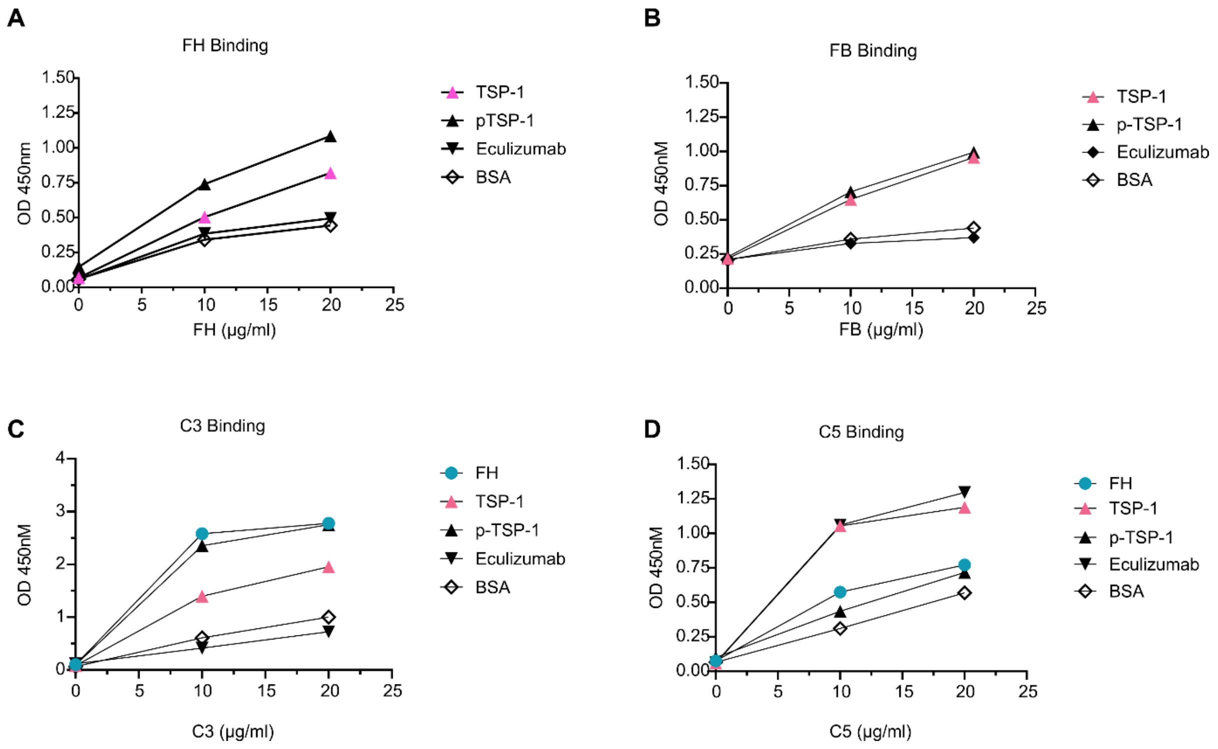


**Figure S1: Recombinant as well as platelet isolated TSP-1 binds to central alternative pathway proteins.** Recombinant TSP-1 (TSP-1), platelet-isolated TSP-1 (p-TSP-1), Eculizumab, BSA or FH were coated on microtiter plates at equimolar concentrations (133 nM). Complement proteins FH, FB, C3 or C5 were added to the wells at indicated concentrations and the amount of bound protein determined via ELISA. **(A)** Binding of TSP-1 and p-TSP-1 to complement protein FH. **(B)** Binding of TSP-1 and p-TSP-1 to complement protein FB. **(C)** Binding of TSP-1 and p-TSP-1 to complement protein C3. **(D)** Binding of TSP-1 and p-TSP-1 to complement protein C5. Results are shown as means.

**Supplemental Figure 2: TSP-1 has no intrinsic decay acceleration or cofactor activity and does not influence FH decay acceleration, FH cofactor activity or FH C3 convertase activity**

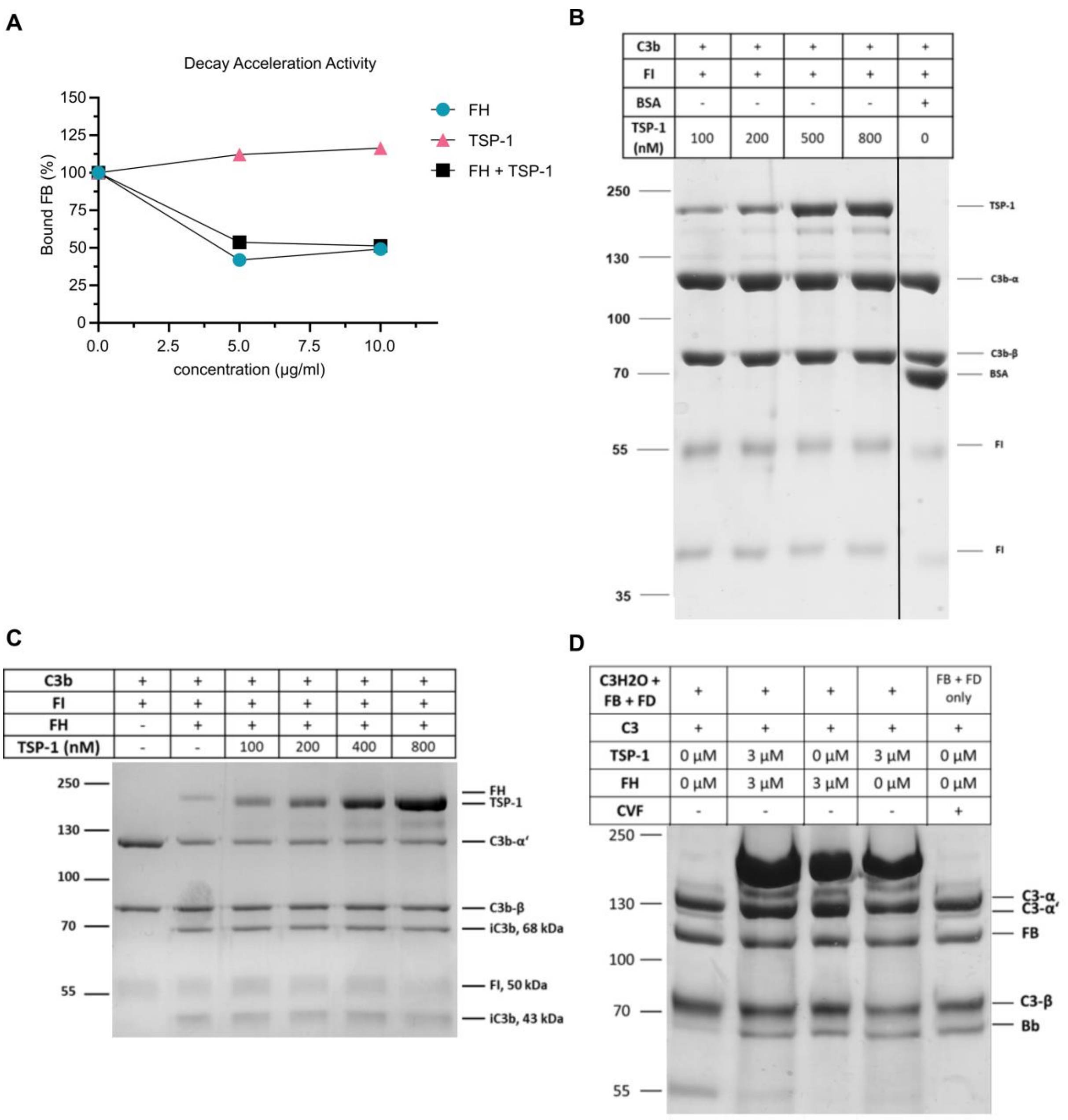


**Figure S2: TSP-1 has no intrinsic decay acceleration or cofactor activity and does not influence FH decay acceleration, FH cofactor activity or FH C3 convertase activity.**

**(A)** TSP-1 does not exhibit decay acceleration activity and does not affect the decay acceleration activity of FH. Microtiter wells were coated with C3b, and C3 convertase was generated by adding FB and FD. Convertases were incubated with increasing concentrations of either FH alone, FH in the presence of 10 µg/ml TSP-1, or increasing concentrations of TSP-1 alone. The amount of intact C3bBb molecules remaining on the surface was detected with

antibodies against FB. Results are shown as the mean of three independent experiments. **(B)** TSP-1 does not display cofactor activity. C3b and FI were incubated with increasing concentrations of TSP-1 or BSA. Cleavage of C3b was visualized on Coomassie-stained SDS gels. A representative image from three independent experiments is shown. Lanes were run on the same gel but were noncontiguous. **(C)** TSP-1 does not affect the cofactor activity of FH. C3b and FI were incubated with 10 nM FH and increasing concentrations of TSP-1. A representative image from three independent experiments is shown. **(D)** TSP-1 does not influence C3 convertase activity of FH. C3($H_2O$) was generated by incubating C3 with 200 mM methylamine for 30 minutes at 37°C. The convertase was generated by incubating C3($H_2O$) with FB and FD. As a positive control, Cobra Venom Factor was incubated with FB and FD instead of C3($H_2O$). After stopping the reaction, C3 was added to the mixture in combination with TSP-1, FH, or both, and the amount of generated C3α' was visualized by Coomassie staining after SDS-PAGE. A representative image from three independent experiments is shown.

**Supplemental Figure 3: TSP-1 Alphafold 3 predicitions demonstrate interaction between TSP-1 and complement proteins mainly via its type 1 repeats**

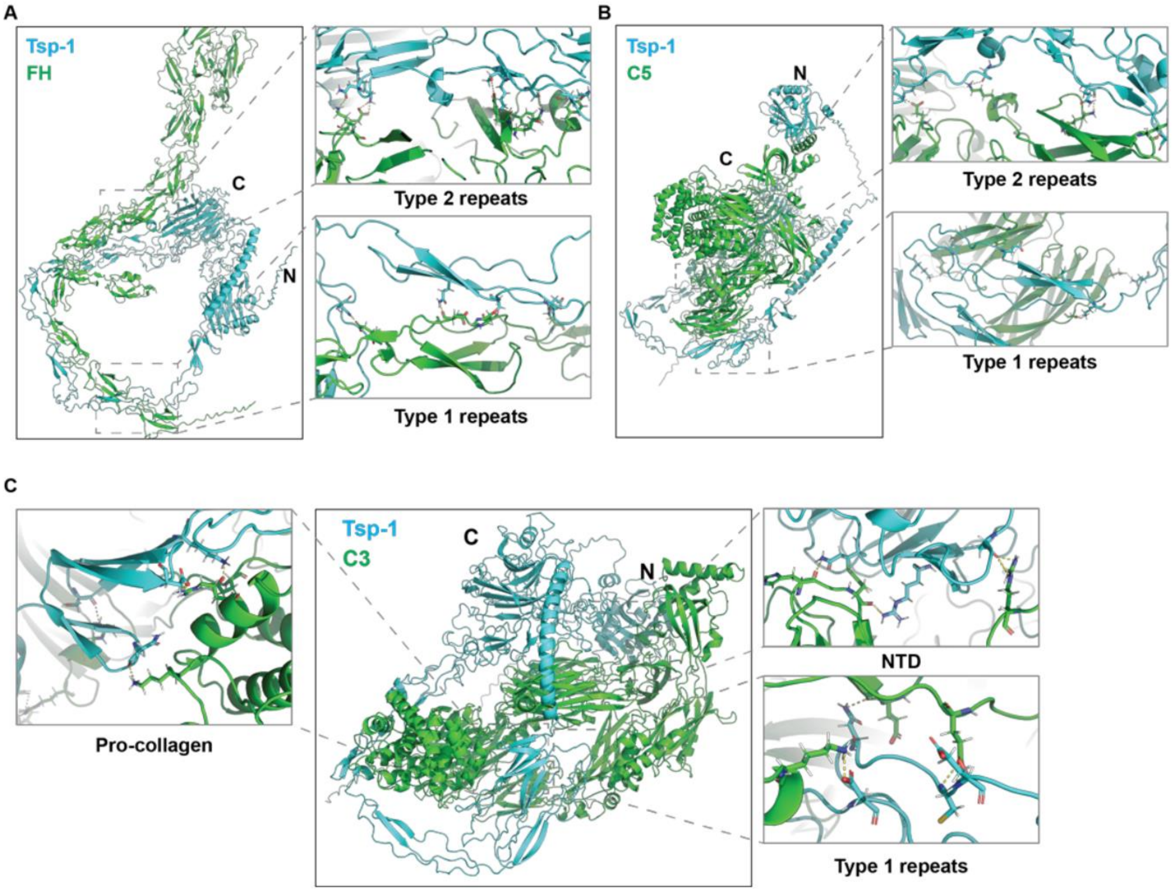


**Figure S3: AlphaFold 3 predictions indicate involvement of TSP-1 type 1 and type 2 repeats in complement interactions.**

Structural models generated by AlphaFold 3 demonstrate interactions between TSP-1 and key proteins of the alternative complement pathway: factor H **(A)**, C5 **(B)**, and C3 **(C)**. Regarding FH and C3, both type 1 and type 2 repeats are predicted to be involved in protein-protein interactions. For C3, additional involvement of the N-terminal domain (NTD), procollagen domain, and type 1 repeats was predicted. NTD – N-terminal domain

**Supplemental Figure 4: TSP-1 protects PNH erythrocytes from complement-mediated opsonization.**

A

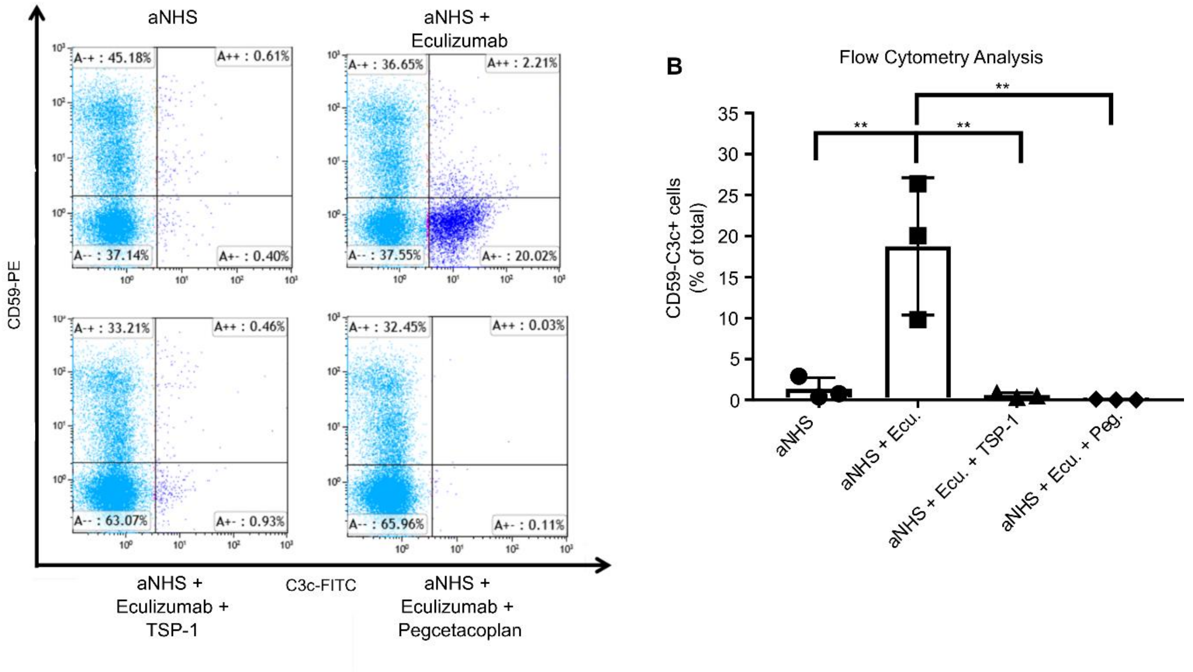


**Supplemental Figure S4: TSP-1 protects PNH erythrocytes from complement-mediated opsonization.**

**(A)** TSP-1 prevents C3 deposition on PNH erythrocytes. PNH Erythrocytes were incubated with acidified serum alone, Eculizumab or with a combination of Eculizumab and TSP-1 or the C3 inhibitor pegcetacoplan. Erythrocytes were stained for CD59 and C3 and percentages of positive and negative stained cells analyzed by flow cytometry. Eculizumab prevents lysis of PNH erythrocytes but leaves C3 depositions on the surface of CD59 negative erythrocytes. Combination of Eculizumab with TSP-1 or pegcetacoplan prevents C3 deposition on CD59 negative PNH erythrocytes. **(B)** Analysis of C3 deposition on PNH erythrocytes from three different patients' samples. Combined treatment of erythrocytes with eculizumab and TSP-1 or eculizumab and pegcetacoplan significantly reduced the amount of C3 positive CD59 negative cells compared to Eculizumab treatment alone. Bars represent means ± SD of 3 independent experiments, **P ≤ 0.01, One-way ANOVA with Tukey's post hoc test. aNHS – acidified normal human serum. Ecu. – Eculizumab; Peg. - Pegcetacoplan

**Supplemental Figure 5: TSP-1 regulates complement on endothelial surfaces as well as in supernatants**

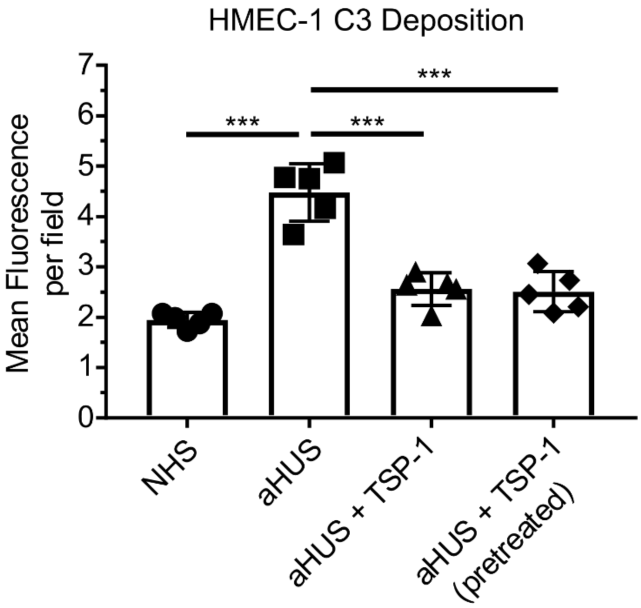


**Figure S5: TSP-1 regulates complement on endothelial surfaces as well as in supernatants.**

HMEC-1 cells were activated with ADP and incubated with 50% normal human serum or aHUS serum with or without 1 µM TSP-1 and stained for C3 deposits. To determine whether TSP-1-mediated complement regulation occurs directly on the endothelial surface, cells were pretreated with 1 µM TSP-1 for 1 hour, washed with PBS, and subsequently stimulated with ADP before adding aHUS serum (aHUS + TSP-1 pretreatment). Pretreatment with TSP-1 protected endothelial cells from pathological C3 deposition similarly to direct addition into aHUS serum. C3 fluorescence intensity was measured in at least 5 randomly chosen high power fields. Results are shown as mean ± SD of 5 independent experiments

**Supplemental Figure 6: Comparison of TSP-1 expression levels between HMEC-1 and HUVEC.**

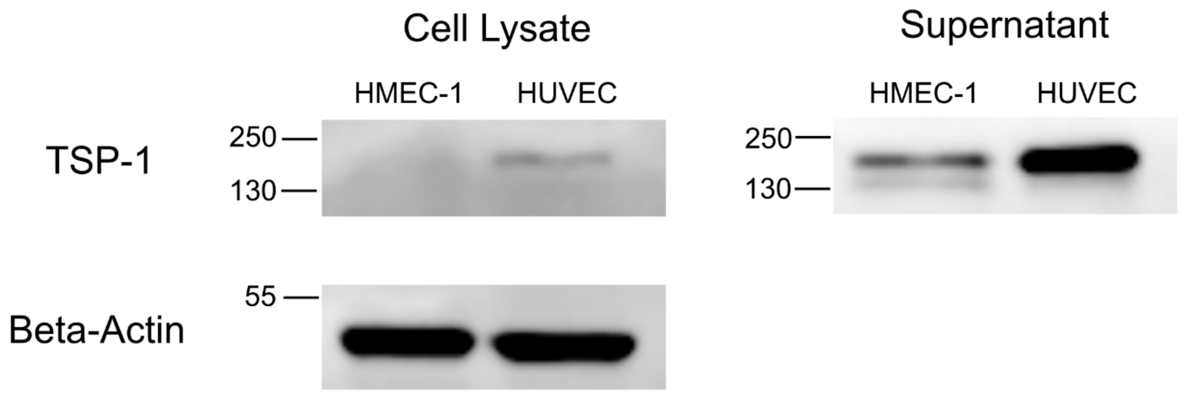


**Figure S6: Comparison of TSP-1 expression levels between HMEC-1 and HUVEC.**

HMEC-1 and HUVEC cells were cultured to 80% confluence and supernatants as well as cell lysates collected for TSP-1 analysis by Western blot. In cell lysates, TSP-1 was undetectable in HMEC-1 cells, whereas a faint band was observed in HUVEC lysates. In contrast, TSP-1 was detected in the supernatants of both cell lines, with HUVEC showing markedly higher TSP-1 secretion compared to HMEC-1.

**Supplemental Figure 7: TSP-1 antibody, but not isotype control, induces NET formation in PR3-treated blood.**

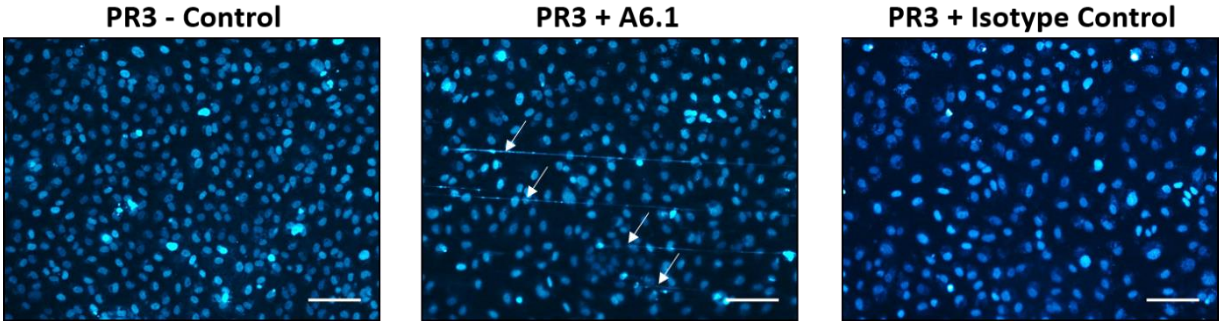


**Figure S7: TSP-1 antibody, but not isotype control, Induces NET formation in PR3-treated blood.** Representative images of live cell imaging in microfluidic chambers performed as described in the Materials and Methods. Hirudinated blood was treated with either PR3 antibodies isolated from AAV patients, PR3 antibodies in combination with a TSP-1 antibody (A6.1), or PR3 antibodies in combination with an isotype control antibody to the TSP-1 antibody. In the PR3-treated samples, no NETs were detected. In contrast, strong NET formation was observed only when PR3 antibodies were combined with the TSP-1 antibody. No NET formation was observed in the isotype control treated samples, demonstrating that the observed effects were due to TSP-1 depletion rather than unspecific effects caused by the antibody type used.

**Supplemental references:**


1. Daubon T, et al. Deciphering the complex role of thrombospondin-1 in glioblastoma development. *Nat Commun*. 2019;10(1):1146.

2. Gaykema LH, et al. Inhibition of complement activation by CD55 overexpression in human induced pluripotent stem cell derived kidney organoids. *Front Immunol*. 2022;13:1058763.

3. Zhu X, et al. Identification of suitable reference genes for real-time qPCR in homocysteine-treated human umbilical vein endothelial cells. *PLoS One*. 2018;13(12):e0210087.

4. Folco EJ, et al. Neutrophil Extracellular Traps Induce Endothelial Cell Activation and Tissue Factor Production Through Interleukin-1α and Cathepsin G. *Arterioscler Thromb Vasc Biol*. 2018;38(8):1901–1912.

5. Lambris JD, et al. A discontinuous factor H binding site in the third component of complement as delineated by synthetic peptides. *J Biol Chem*. 1988;263(24):12147–12150.

6. Michelfelder S, et al. Moss-Produced, Glycosylation-Optimized Human Factor H for Therapeutic Application in Complement Disorders. *J Am Soc Nephrol*. 2017;28(5):1462–1474.

7. Abramson J, et al. Accurate structure prediction of biomolecular interactions with AlphaFold 3. *Nature*. 2024;630(8016):493–500.

8. Yuan X, et al. Small-molecule factor D inhibitors selectively block the alternative pathway of complement in paroxysmal nocturnal hemoglobinuria and atypical hemolytic uremic syndrome. *Haematologica*. 2017;102(3):466–475.
