# Supplementary material for: Thrombospondin-1 inhibits alternative complement pathway activation in antineutrophil cytoplasmic antibody-associated vasculitis
Source: J Clin Invest. 2025 May 8;135(13):e180062. doi: 10.1172/JCI180062 (PMC12208556; doi:10.1172/JCI180062)

Full unedited gel for Figure 2A

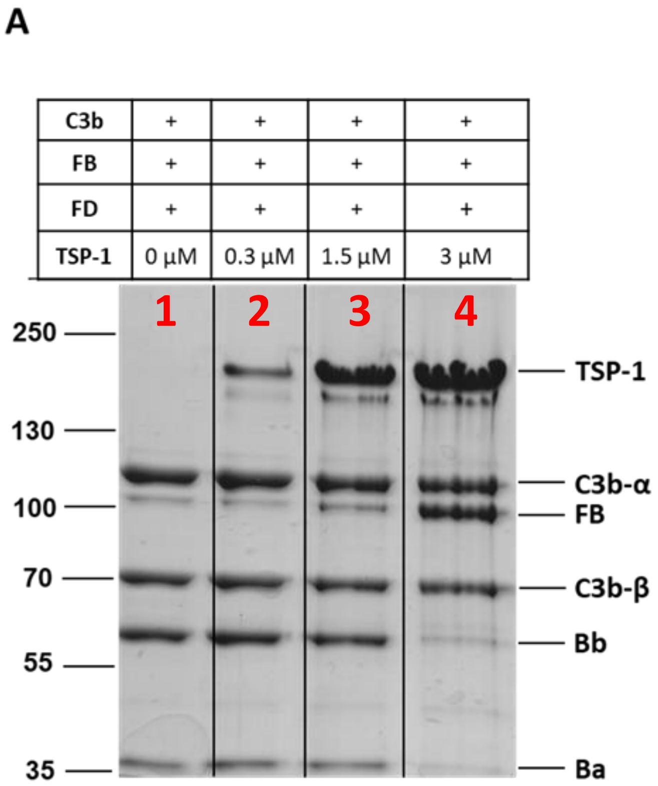

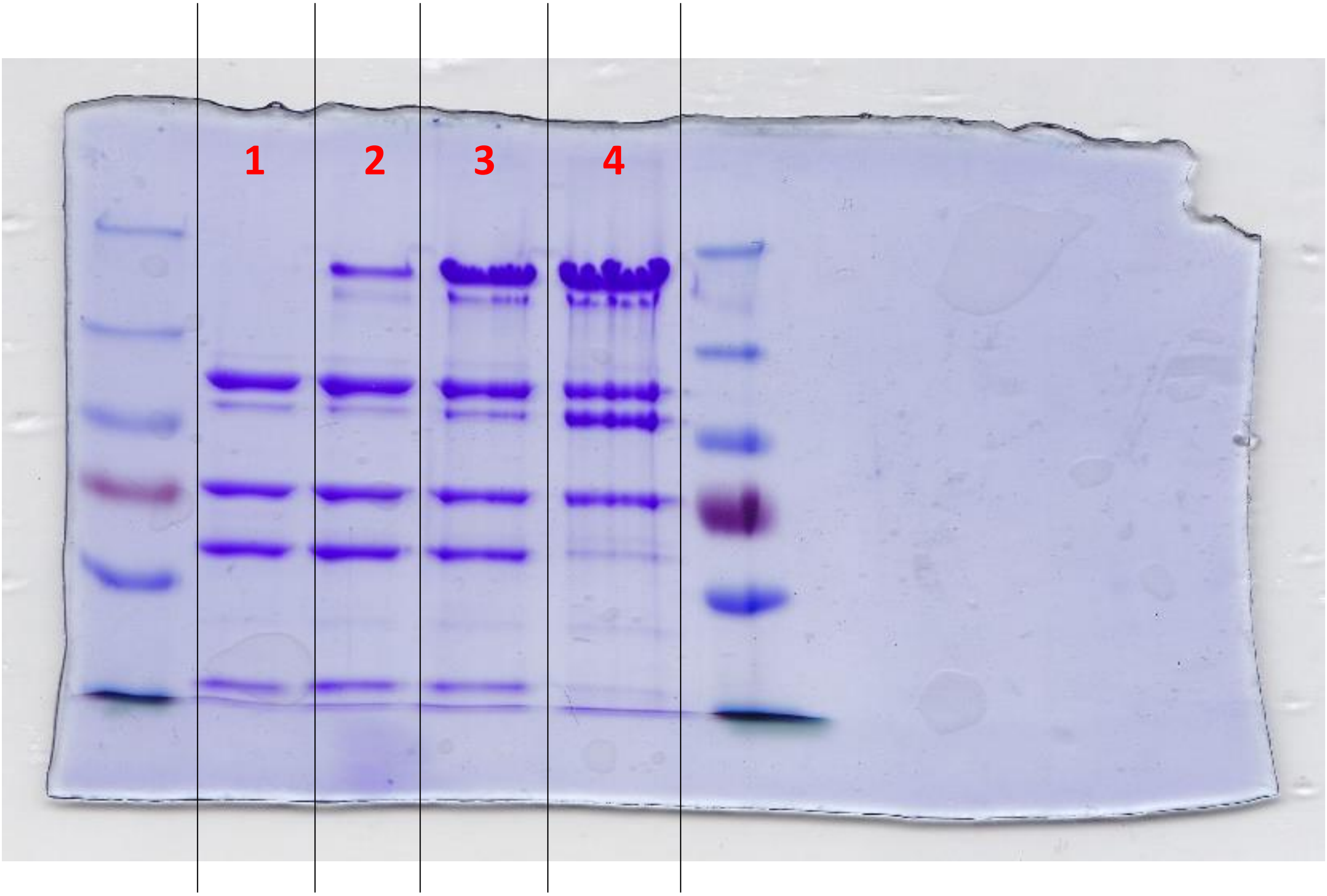

Full unedited gel for Figure 2B

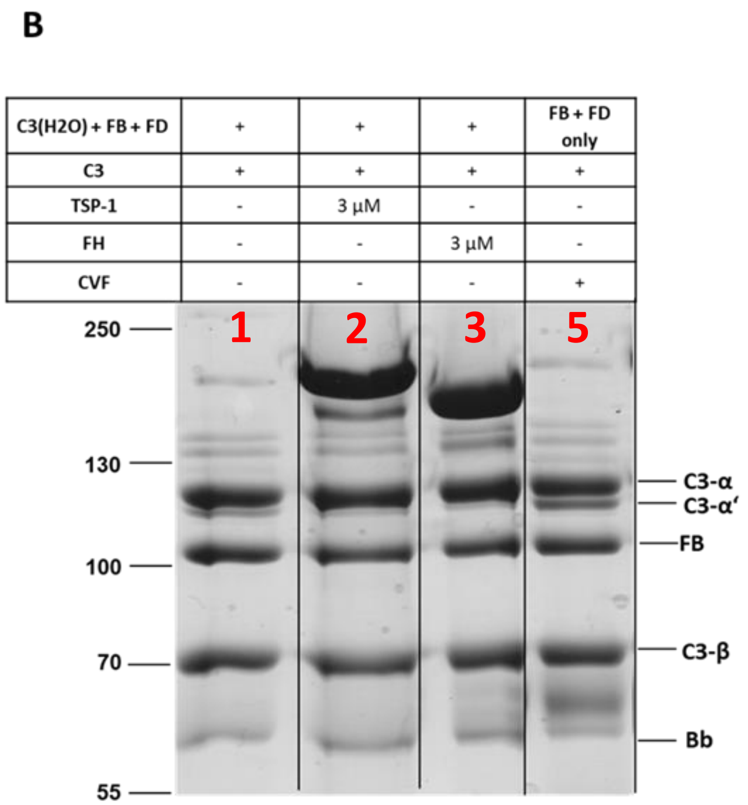

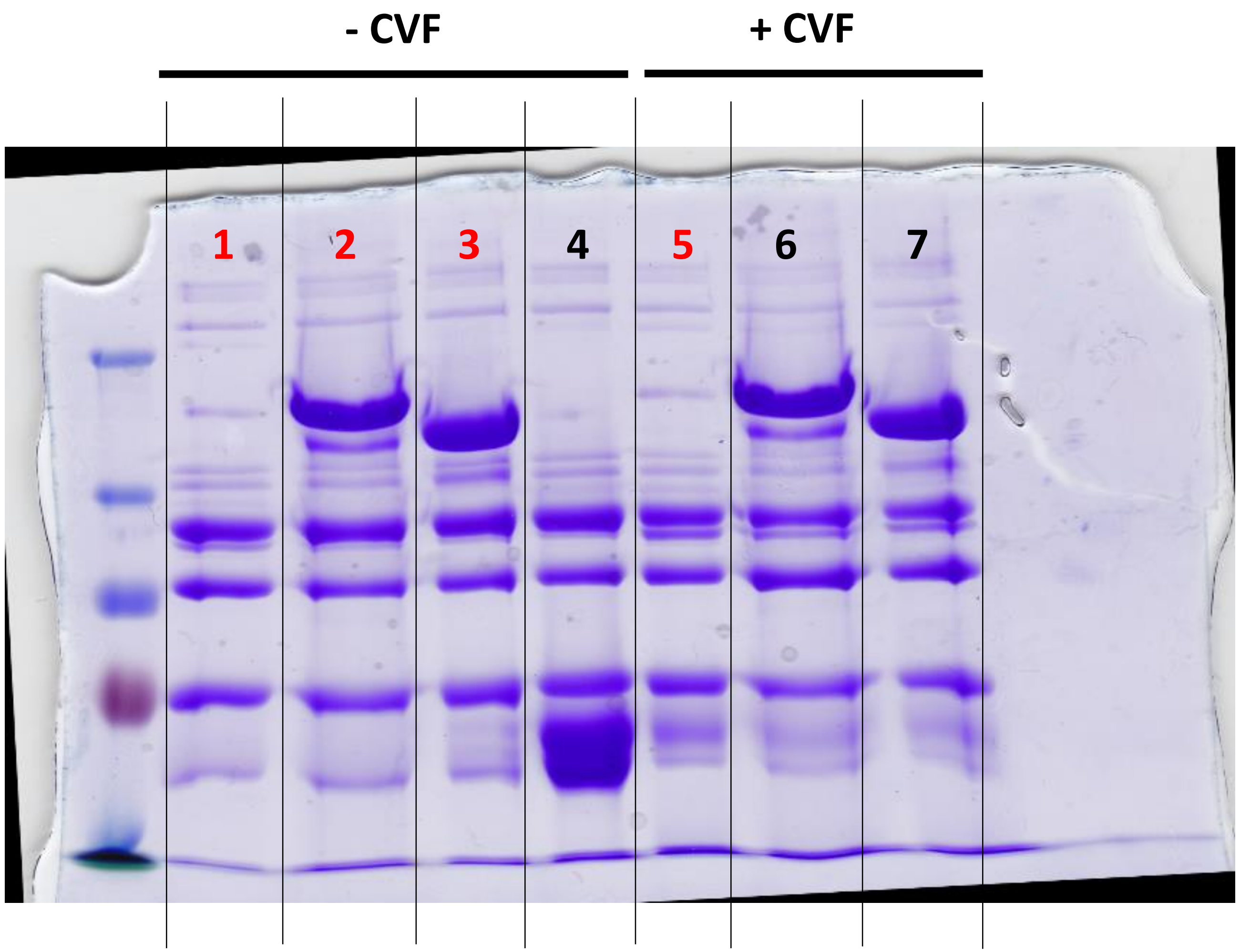

Full unedited gel for supplemental Figure S2B

C3b	+	+	+	+	+
FI	+	+	+	+	+
BSA	-	-	-	-	+
TSP-1 (nM)	100	200	500	800	0

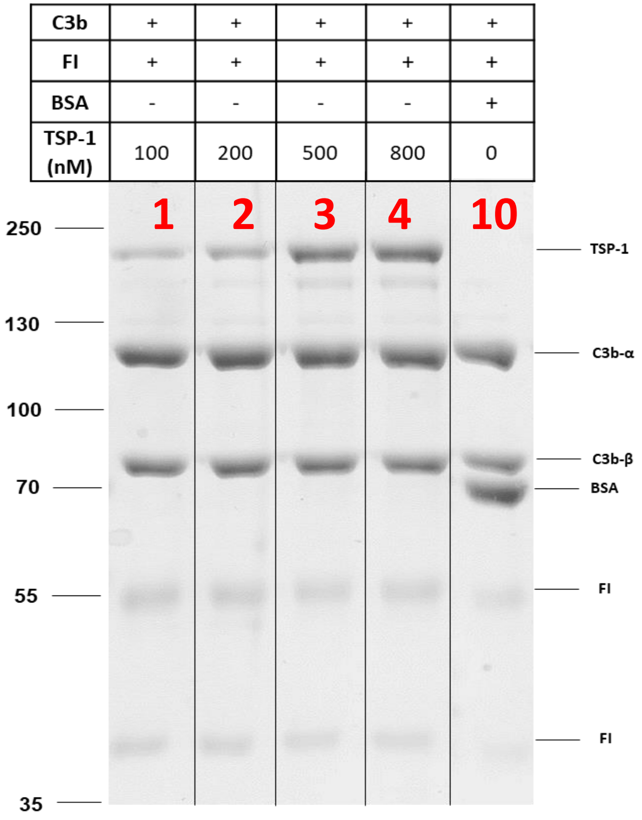

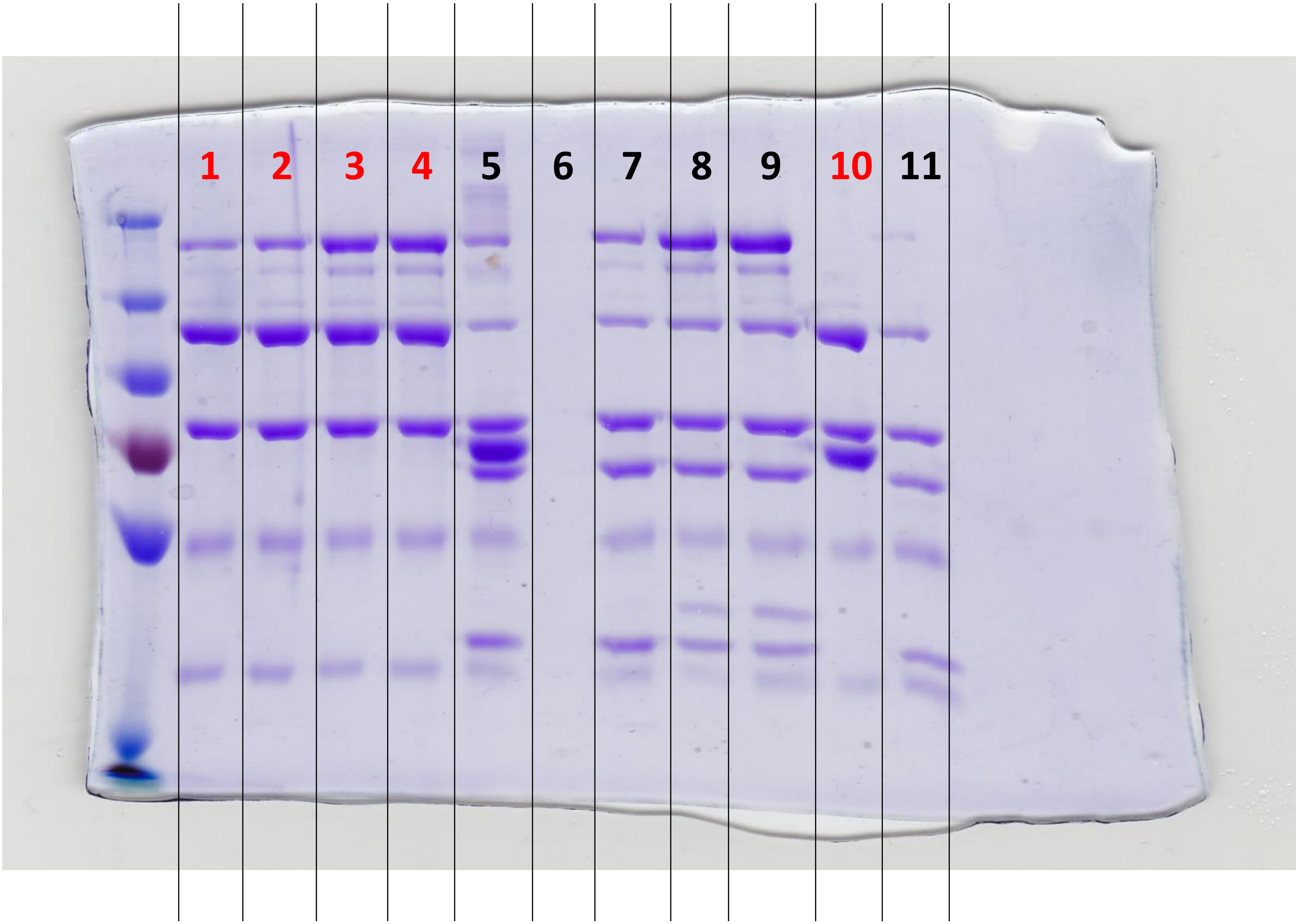

Full unedited gel for supplemental Figure S2C

**C3b**	+	+	+	+	+	+
**FI**	+	+	+	+	+	+
**FH**	-	+	+	+	+	+
**TSP-1 (nM)**	-	-	100	200	400	800

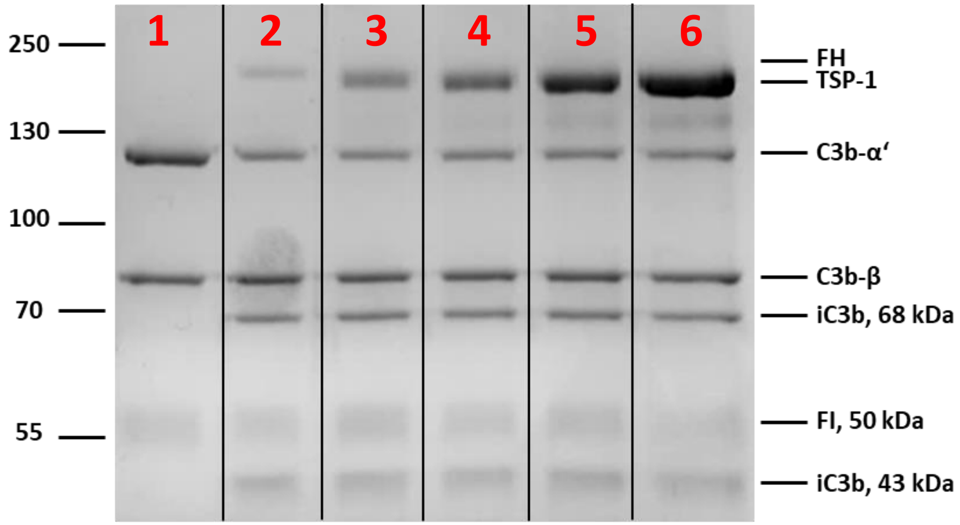

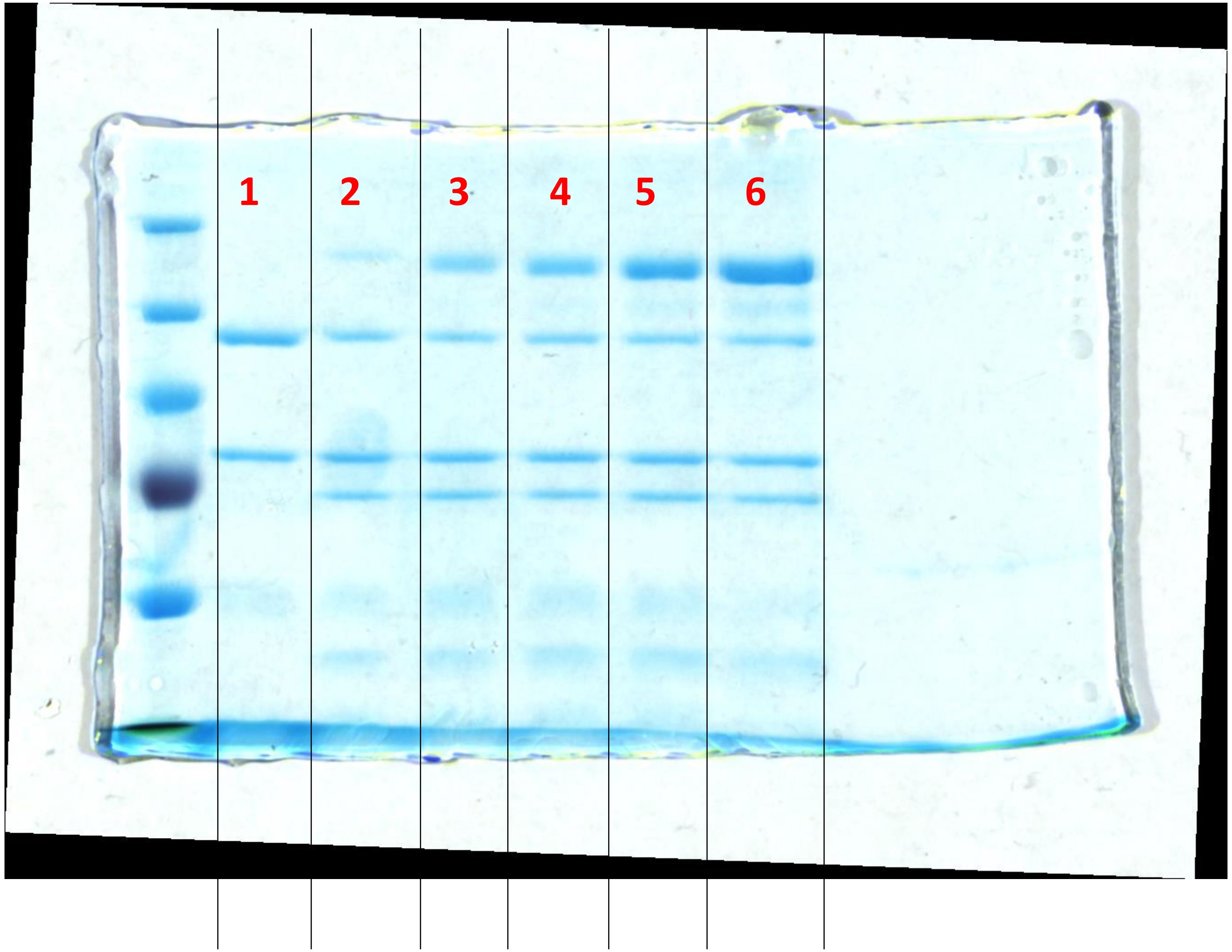

Full unedited gel for supplemental Figure S2D

C3H2O + FB + FD	+	+	+	+	FB + FD only
C3	+	+	+	+	+
TSP-1	0 µM	3 µM	0 µM	3 µM	0 µM
FH	0 µM	3 µM	3 µM	0 µM	0 µM
CVF	-	-	-	-	+

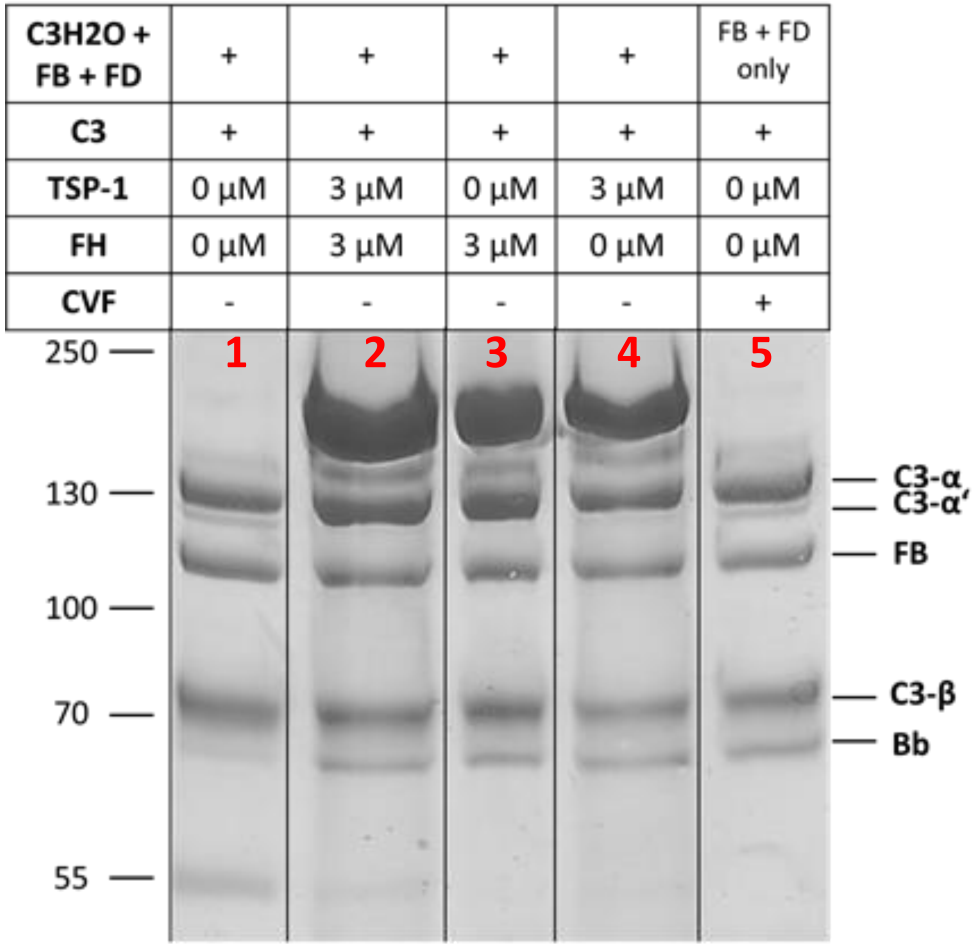

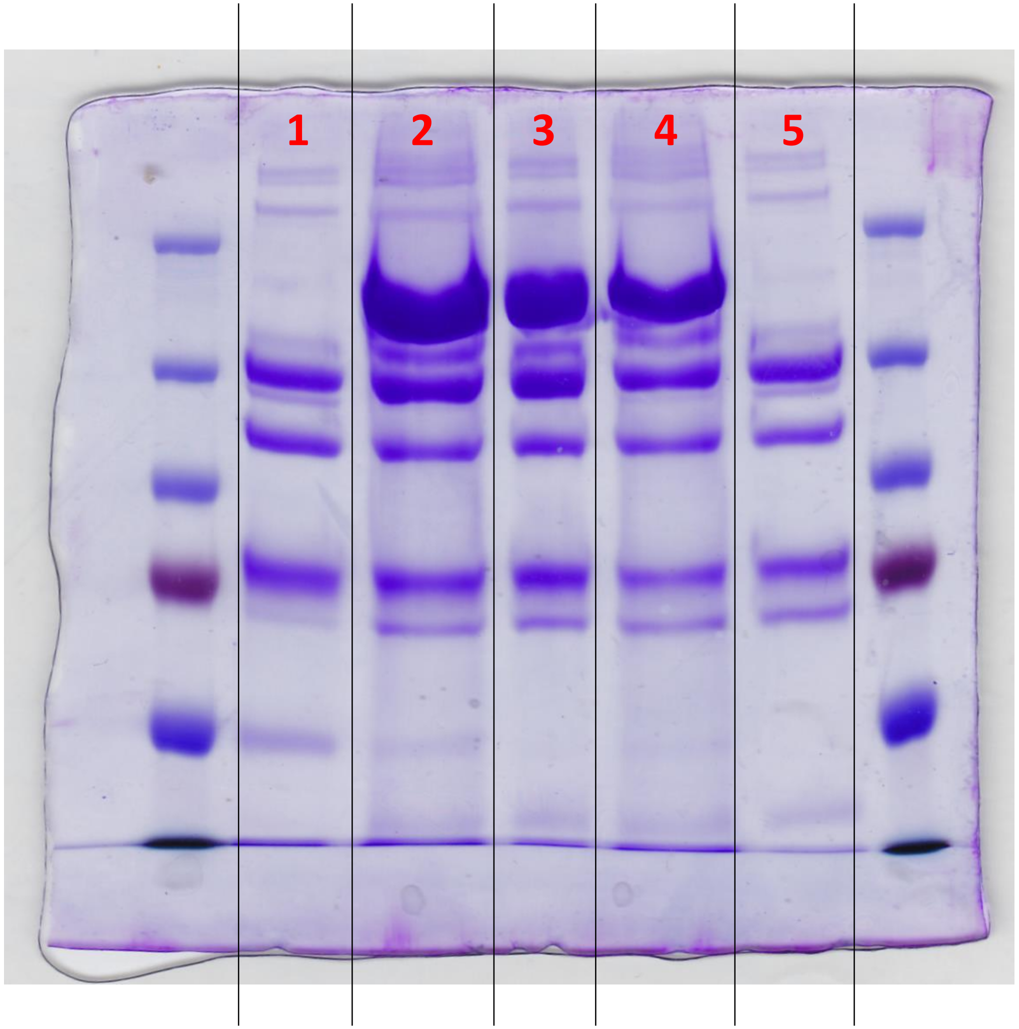

Full unedited blot for supplemental Figure S6

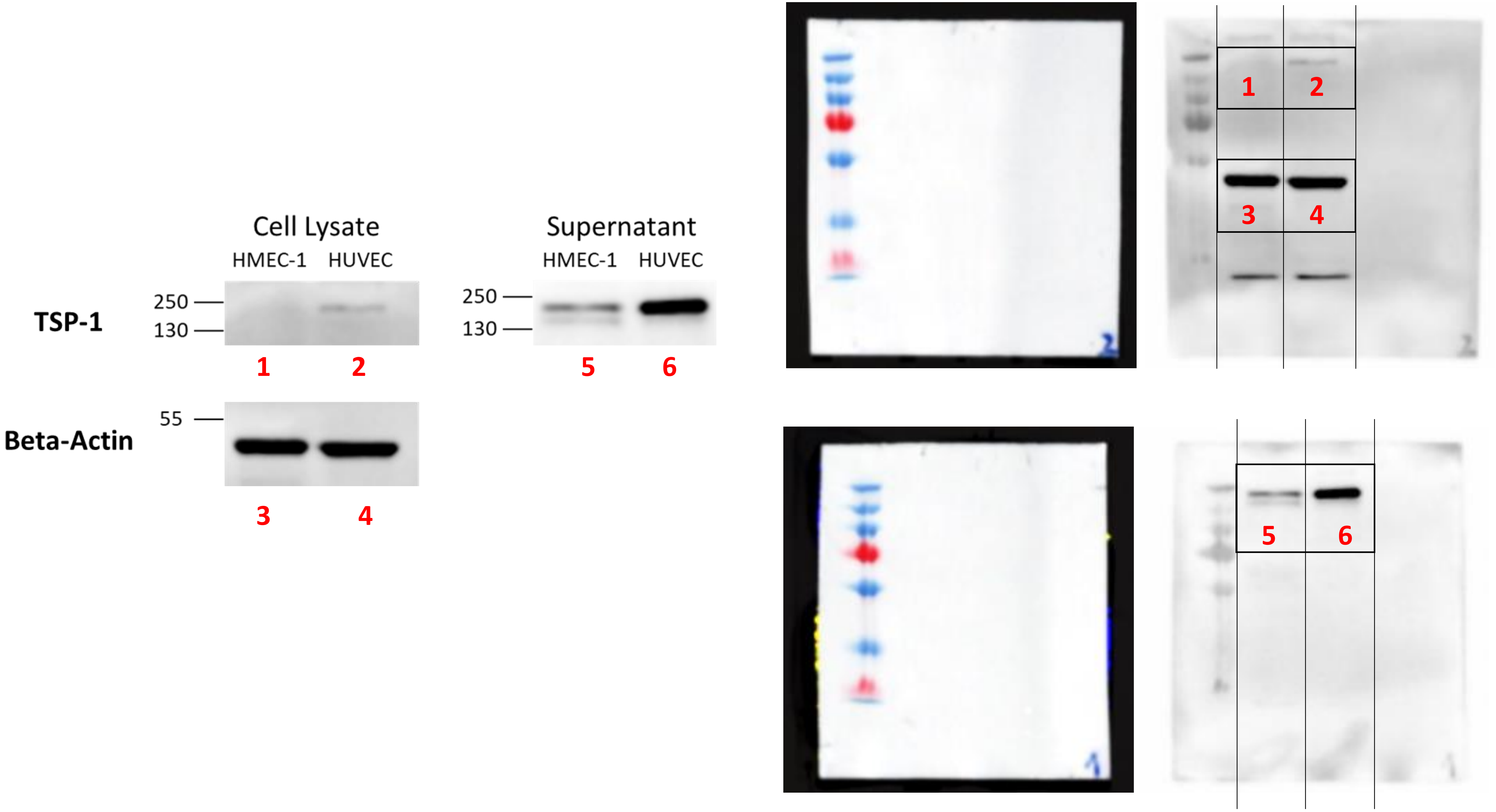

Supplement: Unedited blot and gel images [file jci-135-180062-s113.pdf]
